# An epi‐evolutionary model for predicting the adaptation of spore‐producing pathogens to quantitative resistance in heterogeneous environments

**DOI:** 10.1111/eva.13328

**Published:** 2021-12-31

**Authors:** Frédéric Fabre, Jean‐Baptiste Burie, Arnaud Ducrot, Sébastien Lion, Quentin Richard, Ramsès Djidjou‐Demasse

**Affiliations:** ^1^ INRAE Bordeaux Sciences Agro ISVV SAVE Villenave d’Ornon France; ^2^ IMB Univ. Bordeaux Bordeaux INP CNRS Talence France; ^3^ LMAH Univ. Normandie UNIHAVRE CNRS ISCN Le Havre France; ^4^ CEFE CNRS Univ. Montpellier EPHE IRD Univ. Montpellier 3 Paul‐Valéry Montpellier France; ^5^ MIVEGEC Univ. Montpellier IRD CNRS Montpellier France

**Keywords:** adaptive dynamics, basic reproduction number, integro‐differential equations, quantitative resistance, resistance durability, spore‐producing pathogens

## Abstract

We have modeled the evolutionary epidemiology of spore‐producing plant pathogens in heterogeneous environments sown with several cultivars carrying quantitative resistances. The model explicitly tracks the infection‐age structure and genetic composition of the pathogen population. Each strain is characterized by pathogenicity traits determining its infection efficiency and a time‐varying sporulation curve taking into account lesion aging. We first derived a general expression of the basic reproduction number $R_{0}$ for fungal pathogens in heterogeneous environments. We show that the evolutionary attractors of the model coincide with local maxima of $R_{0}$ only if the infection efficiency is the same on all host types. We then studied the contribution of three basic resistance characteristics (the pathogenicity trait targeted, resistance effectiveness, and adaptation cost), in interaction with the deployment strategy (proportion of fields sown with a resistant cultivar), to (i) pathogen diversification at equilibrium and (ii) the shaping of transient dynamics from evolutionary and epidemiological perspectives. We show that quantitative resistance affecting only the sporulation curve will always lead to a monomorphic population, whereas dimorphism (i.e., pathogen diversification) can occur if resistance alters infection efficiency, notably with high adaptation costs and proportions of the resistant cultivar. Accordingly, the choice of the quantitative resistance genes operated by plant breeders is a driver of pathogen diversification. From an evolutionary perspective, the time to emergence of the evolutionary attractor best adapted to the resistant cultivar tends to be shorter when resistance affects infection efficiency than when it affects sporulation. Conversely, from an epidemiological perspective, epidemiological control is always greater when the resistance affects infection efficiency. This highlights the difficulty of defining deployment strategies for quantitative resistance simultaneously maximizing epidemiological and evolutionary outcomes.

## 1 INTRODUCTION

Resistance to parasites, that is, the capacity of a host to decrease its parasite development (Raberg et al., 2009), is a widespread defense mechanism in plants. Qualitative resistance usually confers disease immunity such that the parasite disease phenotype follows a discrete distribution (“causes disease” vs. “does not cause disease”) on a resistant plant (McDonald & Linde, 2002). Quantitative resistance leads to a decrease in disease severity (Poland et al., 2009; St. Clair, 2010) such that parasite pathogenicity follows a continuous distribution (Lannou, 2012; McDonald & Linde, 2002; St. Clair, 2010). Pathogenicity, also known as aggressiveness, can be estimated in laboratory experiments through the measurement of a number of pathogenicity traits (Lannou, 2012) expressed during the basic steps in the host–pathogen interaction. Quantitative resistance has attracted attention in plant breeding as a means of pathogen control in low‐input cropping systems, particularly due to its supposed higher durability than qualitative resistance (Niks et al., 2015). However, plant pathogens also adapt to quantitative resistance (see Pilet‐Nayel et al. (2017) for a review). The resulting gradual “erosion” of resistance effectiveness (McDonald & Linde, 2002) corresponds, from the pathogen side, to a gradual increase in pathogenicity.

Historically, theoretical studies investigating the effects on pathogen aggressiveness of deploying quantitative resistance in agrosystems have been based on adaptive dynamics (e.g., Gudelj, Fitt, et al. (2004), Gudelj, van den Bosch, et al. (2004), van den Bosch et al. (2006), van den Bosch et al. (2007), van den Berg et al. (2014)). Adaptive dynamics (Dieckmann, 2002; Geritz et al., 1997, 1998) approaches assume that epidemiological and evolutionary processes unfold over different timescales. They essentially focus on long‐term predictions for endemic diseases. Fewer studies (Bourget et al., 2008; Iacono et al., 2012; Rimbaud et al., 2018) have addressed the fundamental short‐ and long‐term objectives of the sustainable management of plant diseases (Rimbaud et al., 2018; Zhan et al., 2015): The short‐term goal is the reduction in disease incidence, whereas the longer‐term objective is to reduce pathogen adaptation to resistant cultivars. Evolutionary epidemiology analysis is well suited to this purpose (Day & Proulx, 2004). Essentially inspired by quantitative genetics, it accounts for the interplay between epidemiological and evolutionary dynamics over the same timescale. As such, it can be used to monitor the dynamics of epidemics and the evolution of any set of pathogen life‐history traits of interest simultaneously. It can also take into account the heterogeneity of host populations resulting, for example, from differences in the genetic, physiological, or ecological states of individuals (Day & Gandon, 2007). This is typically the case with field mixtures, in which several cultivars are cultivated in the same field, and with landscape mosaics, in which cultivars are cultivated in different fields (Rimbaud et al., 2021).

In this article, we follow this approach and study the evolutionary epidemiology of spore‐producing pathogens in heterogeneous agricultural environments. Plant fungal pathogens (*sensu lato*, i.e., including oomycetes) are typical spore‐producing pathogens responsible for nearly one third of emerging plant diseases (Anderson et al., 2004). Spore production is usually a function of the time since infection, due to lesion aging (Kolnaar & Bosch, 2001; Sache et al., 1997; Segarra et al., 2001; van den Bosch et al., 1988). We first formulated a general model explicitly tracking the infection‐age structure and genetic composition of the pathogen population. Mathematically, this model is an extension to heterogeneous plant populations of an integro‐differential model introduced by Djidjou‐Demasse et al. (2017). We then investigated how the deployment of quantitative resistances altering different pathogenicity traits affected pathogen population structure at equilibrium. This question was addressed by highlighting the links between the frameworks of evolutionary epidemiology and adaptive dynamics. We characterized the evolutionary attractors of the coupled epidemiological evolutionary dynamics while emphasizing the differences between the cornerstone concepts of $R_{0}$ in epidemiology (Diekmann et al., 1990; van den Driessche & Watmough, 2008) and invasion fitness in evolution (Dieckmann, 2002; Diekmann et al., 2005; Geritz et al., 1998; Metz et al., 1996; Nowak & Sigmund, 2004). Finally, we investigated the effect of deploying quantitative resistances on the transient behavior of the coupled epi‐evolutionary dynamics, both at epidemiological (disease incidence) and at evolutionary (resistance durability) levels.

## 2 AN EPI‐EVOLUTIONARY MODEL FOR SPORE‐PRODUCING PATHOGENS

Let us suppose that we cultivate two host types differing in terms of quantitative resistance level to a pathogen (e.g., a resistant and a susceptible cultivar). How can we model the joint epidemiological and evolutionary dynamics of the host–pathogen interaction? In this section, we first formulate a general model for the dynamics of spore‐producing plant pathogens in structured populations, which we then apply to the study of specific scenarios.

### 2.1 Host and pathogen populations

Let us consider a spore‐producing plant pathogen infecting a heterogeneous host population with N_c_ host classes. In our study, the host classes are assumed to represent different plant cultivars, but, more generally, host heterogeneity may correspond to differences in host developmental or physiological states or different habitats. At a given time t, hosts in class k can be either healthy $\left(H_{k}\left(t\right)\right)$ or infected. Note that, in keeping with the biology of fungal pathogens, we do not track individual plants, but instead focus on leaf area densities (leaf surface area per m^2^). The leaf surface is viewed as a set of individual patches corresponding to a restricted host surface area that can be colonized by a single pathogen individual (i.e., no co‐infection is possible). Thus, only indirect competition between pathogen strains for a shared resource is considered. Spores produced by all the infectious tissues are assumed to mix uniformly in the air to form a well‐mixed pool of spores that can land on any host class according to the law of mass action. Thus, the probability of contact between a spore and host k is proportional to the total healthy leaf surface area of this host.

The parasite population is structured by a continuous phenotype x and by the time since infection a, such that $I_{k}\left(a,t,x\right)$ represents the density of infected tissue of host k at time t, which have been infected for a duration a with strain x. The density of the airborne pool of spores with phenotype x at time t is denoted by $A\left(t,x\right)$. Both the phenotype and time since infection affect two pathogenicity traits in each host class summarizing the basic steps of the disease infection cycle: (i) infection efficiency $\beta_{k}\left(x\right)$, that is, the probability that a spore deposited on a receptive host surface produces a lesion; and (ii) the sporulation curve $r_{k}\left(a,x\right)$. In line with the biology of plant fungi (Van den Bosch, 1988; Kolnaar & Bosch, 2001; Sache, 1997; Segarra et al., 2001), we assumea gamma sporulation curve defined by four parameters: (i) the latent period $\tau_{k}\left(x\right)$, (ii) the total number of spores $p_{k}\left(x\right)$ produced by a lesion throughout its entire infectious period, and (iii–iv) the rate and shape parameters of the gamma distribution (denoted $\lambda_{k}\left(x\right)$. and $n_{k}\left(x\right)$ respectively). These hypotheses lead to the following sporulation function:

(1) $$r_{k}\left(a,x\right)=\left\{\begin{array}{cc} \frac{p_{k}\left(x\right)\lambda_{k}^{n_{k}\left(x\right)}{\left(a-\tau_{k}\left(x\right)\right)}^{n_{k}\left(x\right)-1}e^{-\lambda_{k}\left(x\right)\left(a-\tau_{k}\left(x\right)\right)}}{\Gamma \left[n_{k}\left(x\right)\right]} & \;\text{if}\;a>\tau_{k}\left(x\right),\\ 0 & \;\text{otherwise}\;. \end{array}\right.$$

where Γ is the gamma function. With this formalism, the duration of the infectious period can be estimated (based on a Gaussian approximation) as $4\sqrt{n_{k}\left(x\right)}/\lambda_{k}\left(x\right)$.

### 2.2 Epi‐evolutionary dynamics

**FIGURE 1:**
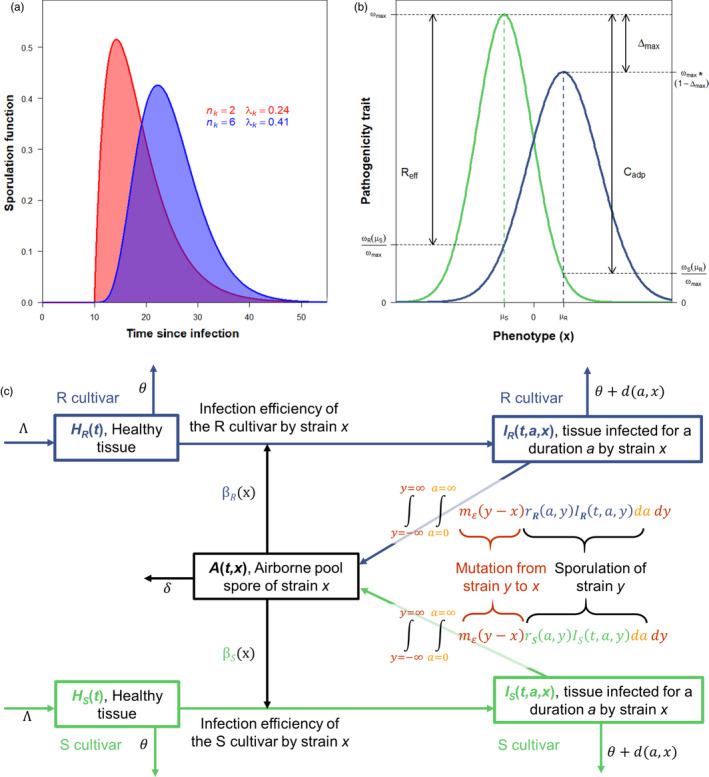
(a) Shapes of gamma sporulation function $r_{k}\left(a,x\right)$ with latent period $\tau_{k}=10$ and total spore production over the entire infectious period $p_{k}=5.94$ as a function of $n_{k}$ and $\lambda_{k}$. The infectious period is ≅24 days for both functions. (b) Phenotypic landscapes of a pathogenicity trait ω on a susceptible (S) and on a resistant (R) cultivar. Trait values are described by unnormalized Gaussian functions. The optimal parasite phenotypes $\mu_{S}$ and $\mu_{R}$ have maximum tras $\omega_{\text{max}}$ and $\omega_{\text{max}}\left(1-\Delta_{\text{max}}\right)$ on the S and R cultivars, respectively. The relative effectiveness of resistance $R_{\text{eff}}=\left(\omega_{S}\left(\mu_{S}\right)-\omega_{R}\left(\mu_{S}\right)\right)/\omega_{\text{max}}$ corresponds to the trait difference between the cultivars of phenotype $\mu_{S}$. The relative cost of adaptation $C_{\text{adp}}=\left(\omega_{S}\left(\mu_{S}\right)-\omega_{S}\left(\mu_{R}\right)\right)/\omega_{\text{max}}$ corresponds to the trait difference between the phenotypes $\mu_{S}$ and $\mu_{R}$ on the S cultivar. Here, $R_{\text{eff}}=0.8$, $C_{\text{adp}}=0.9$, and $\Delta_{\text{max}}=0.2$. **C** Schematic representation of the model with 2 hosts (a susceptible (S) and a resistant (R) cultivar) and a one‐dimensional mutation kernel (i.e., $N_{c}=2$ and N=1). All state variables and parameters are listed in Table 1

With these assumptions, we can derive the following integro‐differential equations to describe the epidemiological and evolutionary dynamics of the host and pathogen populations (see Figure 1C for a schematic representation of the model with 2 hosts):

(2) $$\left(\begin{array}{c} \frac{\partial }{\partial t}H_{k}\left(t\right)=\varphi_{k}\Lambda -\theta H_{k}\left(t\right)-H_{k}\left(t\right)\int_{R^{N}}\beta_{k}\left(y\right)A\left(t,y\right)dy,\\ \left(\frac{\partial }{\partial t}+\frac{\partial }{\partial a}\right)I_{k}\left(t,a,x\right)=-\left(\theta +d_{k}\left(a,x\right)\right)I_{k}\left(t,a,x\right),\\ I_{k}\left(t,0,x\right)=\beta_{k}\left(x\right)H_{k}\left(t\right)A\left(t,x\right),\\ \frac{\partial }{\partial t}A\left(t,x\right)=-\delta A\left(t,x\right)+\sum_{k=1}^{N_{c}}\int_{R^{N}}\int_{0}^{\infty }m_{\varepsilon }\left(y-x\right)r_{k}\left(a,y\right)I_{k}\left(t,a,y\right)da\;dy. \end{array}\right.$$

Healthy hosts are produced at rate Λ and $\varphi_{k}$ is the proportion of the host k at planting in the environment. Healthy hosts can become infected by airborne spores. Thtotal force of infection on a host k is $\int_{R^{N}}\;\beta_{k}\left(y\right)A\left(t,y\right)dy$, where $R^{N}$ is the phenotype space of dimension N. Airborne spores produced by infected hosts become nonviable at rate δ. Healthy hosts die at rate θ (regardless of their class), and infected hosts die at rate $\theta +d_{k}\left(a,x\right)$, where $d_{k}\left(a,x\right)$ is disease‐induced mortality. Hosts infected with strain y produce airborne spores with phenotype x at rate $m_{\varepsilon }\left(y-x\right)r_{k}\left(a,y\right)$, where $m_{\varepsilon }\left(y-x\right)$ is the probability of mutation from phenotype y to phenotype x. Thus, mutations randomly displace strains in the phenotype space at each infection cycle (i.e., generation) according to a mutation kernel $m_{\varepsilon }$. A centered multivariate Gaussian distribution with standard deviation ε is typically used hereafter. However, other mutation kernels can be used provided that they satisfy some general criteria (Appendix S2).

In the simplest scenarios, the phenotype space can be one‐dimensional (i.e., N=1), but we can also consider a multidimensional phenotype space (i.e., N≥2). We refer the reader to Table 1 for a list of the variables and parameters of the model, and to Appendix S3 for a discussion of how to recover several simpler models in the literature from model (2).

**TABLE 1:** Main notations, state variables, and parameters of the model. The general model is structured by time since infection *a*, pathogen strain *x*, and host class *k*. Some of its parameters are functions of these structuring classes

Category	Description	Unit
Notations		
$k\in 1,2,\cdots ,N_{c}$	Index on host class ($N_{c}\geq 2$ class)	
$R^{N}$	Phenotype space of dimension N	
$x\in R^{N}$	Label of the pathogen strain	
a	Time since infection	
States variables		
$H_{k}\left(t\right)$	Density of healthy tissue of host k at time t	
$I_{k}\left(t,a,x\right)$	Density of infected tissue of host k at time t, which have been infected for a duration a with strain x	
$A\left(t,x\right)$	Density of airborne pool of pathogen spores of strain x at time t	
Parameters		
Λ	Birth rate of healthy host tissue	HTD · Tu^−1^
θ	Death rate of host tissue	Tu^−1^
δ	Rate at which spore becomes nonviable	Tu^−1^
$d_{k}\left(a,x\right)$	Disease‐induced mortality of host k after a time units of infection by strain x	Tu^−1^
$\beta_{k}\left(x\right)$	Infection efficiency of pathogen strain x on host k	SD^−1^ · Tu^−1^
$p_{k}\left(x\right)$	Total number of spores produced by a lesion caused by strain x on host k	SD · HTD^−1^
$\tau_{k}\left(x\right)$	Latent period of pathogen strain x on host k	Tu
$\lambda_{k}\left(x\right)$	Rate of the gamma sporulation curve shape for strain x on host k	Unitless
$n_{k}\left(x\right)$	Shape of the gamma sporulation curve shape for strain x on host k	Unitless
$\varphi_{k}$	Proportion of host k at planting	Unitless
ε	Standard deviation of the Gaussian mutation kernel	Unitless

Note

Abbreviations: HTD, host tissue density; SD, spore density; and Tu, time unit.

## 3 BASIC REPRODUCTION NUMBER AND INVASION FITNESS

Model (2) allows us to track the coupled epidemiological and evolutionary dynamics of the host–pathogen interaction. We can use a numerical integration of this model to determine the transient dynamics toward potential evolutionary attractors. For further analytical progress, we can consider two limiting cases of this process. First, in an initially uninfected population, whether or not a single pathogen strain spreads in the population can be determined by calculating the basic reproduction number of this strain. Second, the long‐term evolutionary endpoints can be determined by calculating the invasion fitness of a rare mutant in a resident population at equilibrium according to the standard adaptive dynamics methodology.

### 3.1 Basic reproduction number: Invasion in an uninfected population

The basic reproduction number, usually denoted $R_{0}$, is defined as the total number of infections arising from one newly infected individual introduced into a healthy (disease‐free) host population (Anderson, 1991; Diekmann et al., 1990). It is typically used to study the spread of a pathogen strain x in an uninfected host population. In an environment with N_c_ host classes in which all the pathogen propagules pass through a common pool in compartment *A* as in model (2), $R_{0}$ is the sum of pathogen reproduction numbers for each host class (Rueffler & Metz, 2013). A pathogen with phenotype *x* will spread if $R_{0}\left(x\right)>1$, with

(3) $$R_{0}\left(x\right)\;=\;\sum_{k=1}^{N_{c}}R_{0}^{k}\;\left(x\right)$$

where the quantity $R_{0}^{k}\left(x\right)=\Psi_{k}\left(x\right)H_{k}^{0}$ is the basic reproduction number of a pathogen with phenotype x in host k. This expression depends on the disease‐free equilibrium density of healthy hosts $H_{k}^{0}=\varphi_{k}\Lambda /\theta$ and $\Psi_{k}\left(x\right)$, the reproductive value of a pathogen with phenotype x landing on host k (i.e., the fitness function, Appendix S4). It is given by

(4) $$\psi_{k}\left(x\right)=\frac{1}{\delta }\beta_{k}\left(x\right)\int_{0}^{\infty }r_{k}\left(a,x\right)\underset{\text{prob}\text{.that}\;a\;\text{lesion}\;\text{is}\;\text{viable}\;\text{at}\;\text{age}\;a}{\underbrace{\exp(-\theta a-\int_{0}^{a}d_{k}\left(\sigma ,x\right)d\sigma )}}da,$$

In this expression, multiplying the probability that a lesion is viable at time since infection a by $r_{k}\left(a,x\right)$ and integrating over all times since infection a gives the total number of spores effectively produced by a lesion during its lifetime. This differs from $\left(x\right)$, which is the number of spores potentially produced if the lesion remains viable during the whole infectious period. A closely related expression with a simpler model (single host type and no disease‐induced mortality) was derived by Gilchrist et al. (2006).

Assuming that *d_k_* does not depend on the time since infection and a gamma sporulation function (1), we obtain

(5) $$\Psi_{k}\left(x\right)=\frac{1}{\delta }\times \beta_{k}\left(x\right)\times e^{-\left(\theta +d_{k}\left(x\right)\right)\tau_{k}\left(x\right)}\times p_{k}\left(x\right)\times {\left(\frac{\lambda_{k}}{\lambda_{k}+\theta +d_{k}\left(x\right)}\right)}^{n_{k}}.$$

Furthermore, if $r_{k}$ is a constant (i.e., $r_{k}\left(a,x\right)=p_{k}\left(x\right)$ and $d_{k}\left(a,x\right)=d_{k}\left(x\right)$ for all a), we recover the classical expression of $R_{0}$. for SIR models (Day, 2002) with

$$\Psi_{k}\left(x\right)=\frac{\beta_{k}\left(x\right)p_{k}\left(x\right)}{\delta \left(\theta +d_{k}\left(x\right)\right)}.$$

### 3.2 Invasion fitness and long‐term evolutionary attractors

Once a pathogen strain x has reached an endemic equilibrium, invasion by a new mutant strain can be studied with the adaptive dynamics methodology. A rare mutant strain with phenotype y will invade a resident pathogen population with phenotype x if its invasion fitness $f_{x}\left(y\right)>0$. Potential evolutionary endpoints can be identified as the solutions of $f_{x}\left(y\right)>0$. The sign of this two‐dimensional function is classically visualized on a pairwise invasibility plot (PIP) (Dieckmann, 2002; Diekmann et al., 2005; Geritz et al., 1997, 1998; Metz et al., 1996; Nowak & Sigmund, 2004). In our model, the existence of a common pool of pathogen propagules allows us to write (Appendix S6) the invasion fitness $f_{x}\left(y\right)$ as

(6) $$f_{x}\left(y\right)=\sum_{k=1}^{N_{c}}\underset{\text{feed}\;\text{back}\;\text{of}\;\text{resident}\;x}{\underbrace{\frac{\Lambda \varphi_{k}}{\theta +\beta_{k}\left(x\right)A^{x}}}}\left(\psi_{k}\left(y\right)-(\psi_{k}\left(x\right)\right).$$

Environmental feedback of the resident strain x conditions the ability of a mutant strain *y* to invade the resident population. It depends on the conditions established by the resident strain, particularly as concerns the resources of host k already taken by x. When infection efficiencies do not differ between host classes (i.e., $\beta_{k}=\beta$, for all host classes k), this feedback term is unique and comes out of the sum, and using equation (3), equation (6) can be rewritten as

(7) $$f_{x}\left(y\right)=\frac{\theta }{\theta +\beta \left(x\right)A^{x}}\left(R_{0}\left(y\right)-R_{0}\left(x\right)\right).$$

It follows that model (2) admits an optimization principle based on $R_{0}$ (Gyllenberg, & Service, 2011; Lion & Metz, 2018; Mylius & Diekmann, 1995; Metz et al., 2008; Rueffler & Metz, 2013). Indeed, the sign of the invasion fitness $f_{x}\left(y\right)$ is given by the sign of the difference between $R_{0}\left(y\right)$ and $R_{0}\left(x\right)$; and thus, the evolutionary attractors of model (2) coincide with the local maxima of $R_{0}$ provided that host plants affect only sets of pathogenicity traits underlying sporulation curves. Conversely, if infection efficiencies differ for at least two host classes, the optimization principle does not apply. Accordingly, if some plant hosts affect infection efficiency, the calculation of invasion fitness with equation (6) is required for the characterization of evolutionary attractors.

## 4 CASE STUDY: DEPLOYMENT OF QUANTITATIVE RESISTANCES

As an application of our general model, we now consider two habitats corresponding to a susceptible (S) and a resistant (R) cultivar differing by a single quantitative resistance trait. We assume that, after a long period of monoculture of the S cultivar, a fraction φ of the S cultivar is replaced by the R cultivar at *t* = 0. We consider two scenarios, depending on how the resistance trait affects the life cycle of the pathogen. In the SP scenario, the resistance affects only total spore production, whereas in the IE scenario, the resistance affects only infection efficiency. The analysis of the scenarios makes use of analytical results from adaptive dynamics and simulation results from evolutionary epidemiology while highlighting the links between these frameworks.

### 4.1 Scenario simulations and model outputs

*Phenotypic landscapes on the S and R cultivars*. Model simulation first requires the specification of fitness functions describing the pathogenicity trait values of any strain x on each cultivar (Figure 1B). For a trait ω (either total spore production in the SP scenario, or infection efficiency in the IE scenario), we used the unnormalized Gaussian function

$$\omega_{s}\left(x\right)=\omega_{\max}\times \left(\exp-\frac{{\left(x+\mu \right)}^{2}}{2\sigma_{S}^{2}}\right)\;\text{and}\;\omega_{R}\left(x\right)=\omega_{\max}\left(1-\Delta_{\max}\right)\times \exp-\left(\frac{{\left(x-\mu \right)}^{2}}{2\sigma_{R}^{2}}\right).$$

Without loss of generality, and to simplify the presentation, the optimal phenotypes are opposite for the S and R cultivars. Therefore, the optimal parasite phenotypes $\mu_{S}=-\mu$ and $\mu_{R}=\mu$ are characterized by maximal trait values $\omega_{\text{max}}$ and $\omega_{\text{max}}\left(1-\Delta_{\text{max}}\right)$ on the S and R cultivars, respectively, where $\Delta_{\text{max}}$ is the relative difference in maximal traits between the two cultivars. The inverse of the variances $1/\sigma_{S}^{2}$ and $1/\sigma_{R}^{2}$ defines the selectivity of each habitat. Rather than using these parameters, the phenotypic landscapes can be reparameterized to fit the terminology used in plant pathology to describe quantitative resistances with the relative effectiveness of resistance $R_{\text{eff}}$ and the relative cost of adaptation $C_{\text{adp}}$ (Figure 1B). More precisely, for a given $\sigma_{S}>0$, $R_{\text{eff}}$ and $C_{\text{adp}}$ both in $[0,1[$ and $\Delta_{\text{max}}$ in $[0,R_{\text{eff}}[$, we have

(8) $$\frac{1}{\sigma_{R}^{2}}=\frac{1}{\sigma_{S}^{2}}\frac{\log\left(1-R_{\text{eff}}\right)-\log\left(1-\Delta_{\text{max}}\right)}{\log(1-C_{\text{adp}})}\;\text{and}\;\mu =\sigma_{s}\left[-0.5\log(1-C_{\text{adp}})\right].$$

**FIGURE 4:**
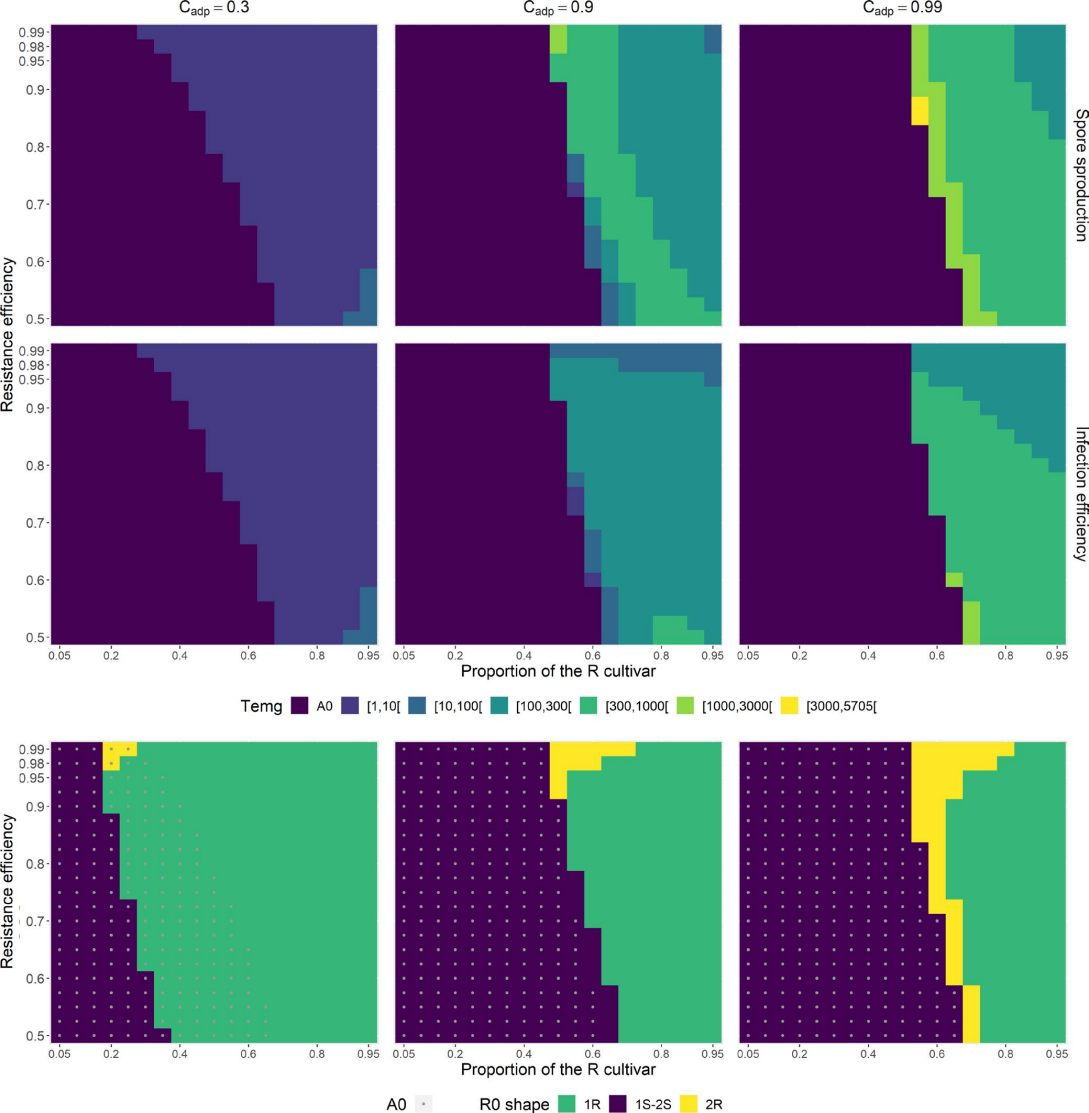
Time to emergence of the evolutionary attractor. The time to emergence *T*_emg_ is the time at which the proportion of the evolutionary attractor (the closest to the optimal phenotype on the R cultivar) becomes ≥5% in the pool of spores in the air. **Line 1**. The resistance affects only total spore production (SP scenario). *T*_emg_ is characterized as a function of the proportion of the R cultivar at planting (*x*‐axis) and of the relative effectiveness of the resistant cultivar (*y*‐axis) for three costs of adaptation (columns). Level A0 indicates situations in which the criteria for emergence are met right from the initial conditions. **Line 2**. An in line 1 when the resistancaffects only infection efficiency (IE scenario). **Line 3**. Shape of the $R_{0}$ function. Three shapes are classified: (i) "1S‐2S" when the evolutionary attractor $\mu^{*}$ is closer to $\mu_{S}$ than to $\mu_{R}$ (*i*.*e*., $\mu^{*}<0$), (ii) "1R" when $\mu^{*}$ is closer to $\mu_{R}$ and $R_{0}$ is unimodal, and (iii) "2R" when $\mu^{*}$ is closer to $\mu_{R}$ and $R_{0}$ is bimodal. Point A0 indicates situations in which the criteria for emergence are met right from the initial conditions. For all panels, all the other parameters are set to their reference values (Table 2)

*Parameter values and initial conditions*. For each scenario, simulations were used to explore how the relative effectiveness of resistance *R*_eff_ (between 0.5 and 0.99), the relative cost of adaptation $C_{\text{adp}}$ (between 0.3 and 0.99), and the deployment strategy φ (between 0.05 and 0.95) affect coupled epidemiological and evolutionary dynamics. Note that, within the parameter space considered, the trade‐off function that links $\omega_{S}$ and $\omega_{R}$ in both cultivars can be concave, convex, or sigmoidal (Figure S1). Similarly, the basic reproduction ratio $R_{0}$ can have either one global maximum, or one global and one local maximum, and this global maximum can be closer to $\mu_{S}$ or to $\mu_{R}$ (Figure 4, line 3). These features strongly affect the dynamics. Simulations were initiated with a density of airborne spores $A\left(0,x\right)$ at mutation–selection equilibrium in an environment in which only the S cultivar was grown.

All model parameters and initial conditions are summarized in Table 2. Parameters for pathogenicity traits do not fit a particular pathogen species but are instead typical of biotrophic foliar fungal diseases, such as wheat rusts on susceptible cultivars. Based on Rimbaud et al. (2018), we set the infection efficiency to 0.2 and the duration of the latent and infectious periods to 10 and 24 days, respectively. Total spore production was set so as to obtain an $R_{0}$ of 30 in an environment containing only the S cultivar (Mikaberidze et al., 2016).

**TABLE 2:** Initial conditions and parameters used for simulations with the S and R cultivars. Two scenarios were considered: The R cultivar can alter either the infection efficiency of the pathogen (IE) or its total spore production (SP). The reference values given for each parameter are those used in all simulations, unless otherwise stated. Values in brackets indicate the range of variation [minimum, maximum] used for the numerical exploration

Name	Description	Values
Pathogenicity traits		
$\beta_{\text{max}}$	Maximal infection efficiency on the S cultivar	0.2
$\tau_{\text{max}}$	Latent period associated with maximal sporulation	10
$p_{\text{max}}$	Maximal total spore production on the S cultivar	5.94^a^
n	Shape of the gamma sporulation curve	2
λ	Rate of the gamma sporulation curve	0.24
Pathogenicity landscape for the trait ω affected by the resistance gene		
$R_{\text{eff}}$	Relative effectiveness of the R cultivar	[0.5, 0.99]
$C_{\text{adp}}$	Relative cost of adaptation	[0.3, 0.99]
$\omega_{\text{max}}$	Maximal trait value on the S cultivar	$\beta_{\text{max}}$ (IE)^b^; $p_{\max}$ (SP)^b^
$\Delta_{\text{max}}$	Relative difference of maximal traits between S and R cultivars	0
$1/\sigma_{S}^{2}$	Selectivity of the S cultivar	$0.06^{-2}$
$\omega_{S}\left(x\right)$	Trait value on the S cultivar	$\omega_{\text{max}}f_{N}\left(-\mu ,\sigma_{S},x\right)$ ^c^
$\omega_{R}\left(x\right)$	Trait value on the R cultivar	$\omega_{\text{max}}\left(1-\Delta_{\text{max}}\right)f_{N}\left(\mu ,\sigma_{R},x\right)$ ^c^
Others parameters		
Λ	Birth rate of healthy host tissue	0.35
θ	Death rate of host tissue	0.01
δ	Rate at which spore becomes nonviable	1
ε	Standard deviation of the Gaussian mutation kernel	0.005
d	Disease‐induced mortality	0
φ	Proportion of the R cultivar at planting	[0.05, 0.95]
$T_{\text{dpl}}$	Duration of R cultivar deployment	750
Initial conditions		
$H_{S}\left(0\right)$	Density of healthy tissue on the S cultivar	$\left(1-\varphi \right)\Lambda /\theta$
$H_{R}\left(0\right)$	Density of healthy tissue on the R cultivar	φΛ/θ
$i_{S}\left(0,a,x\right)$	Density of infected tissue on the S cultivar	0
$i_{R}\left(0,a,x\right)$	Density of infected tissue on the R cultivar	0
$A\left(0,x\right)$	Density of the airborne pool of pathogen spores	Mutation–selection equilibrium^d^
Evolutionary and epidemiological outputs		
$T_{\text{emg}}$	Time to emergence of the adapted strain $\mu^{*}$	Time from which, for any $t\geq T_{\text{emg}}$,
		$A\left(t,\mu^{*}\right)/\left(A\left(t,\mu_{S}\right)+A\left(t,\mu^{*}\right)\right)\geq 5\text{\textbackslash{}\%}\;$
rHAD	Relative healthy area duration gain	$\int_{0}^{T_{\text{dpl}}}\left(H_{S}+H_{R}\right)\left(t\right){{|}_{\varphi }dt/\int_{0}^{T_{\text{dpl}}}\left(H_{S}\left(t\right)\right)|}_{\varphi =0}dt$

^a^ *p*_max_ is such that $R_{0}\left(\mu_{S}\right)=30$ for φ=0, leading to $p_{\max}=30\times \theta \delta \exp\left(\theta \tau_{\max}\right)\frac{{\left(1+\theta /\lambda \right)}^{n}}{\left(\beta_{\max}\Lambda \right)}$.

^b^ $\omega_{\text{max}}=\beta_{\text{max}}$ for the IE scenario and $\omega_{\text{max}}=p_{\text{max}}$ for the SP scenario.

^c^ The notations $f_{N}\left(\mu ,\sigma ,x\right)=\exp\left[-{\left(x-\mu \right)}^{2}/\left(2\sigma^{2}\right)\right]$ indicate the unnormalized density function of the Gaussian distribution. The optimal phenotype value μ and the selectivity of the R cultivar $1/\sigma_{R}^{2}$ are calculated from equation (8).

^d^ The density of the airborne pool of pathogen spores at mutation–selection equilibrium is determined by running the model over 3000 generations in an environment containing only the S cultivar.

*Evolutionary and epidemiological outputs*. The evolutionary ouut considered was the time to emergence *T*_emg_ of the adapted strain $\mu^{*}$. This corresponds to the time from which its proportion in the airborne pool of spores relative to $\mu_{S}$ remains ≥0.05 (Table 2). If the equilibrium is monomorphic, the adapted strain $\mu^{*}$ is the only evutionary attractor. If the equilibrium is polymorphic, the adapted strain $\mu^{*}$ is considered as the one closest to $\mu_{R}$. The epidemiological control provided by deployment of the R cultivar was assessed by determining the relative healthy area duration (rHAD) gain, a variable used as a proxy for crop yield (Iacono et al., 2012; Rimbaud et al., 2018). It was calculated over 750 generations by assuming the deployment of the R cultivar over a period of 50 years, with 15 generations of the pathogen per year (Table 2).

### 4.2 Evolutionary outcomes

**FIGURE 2:**
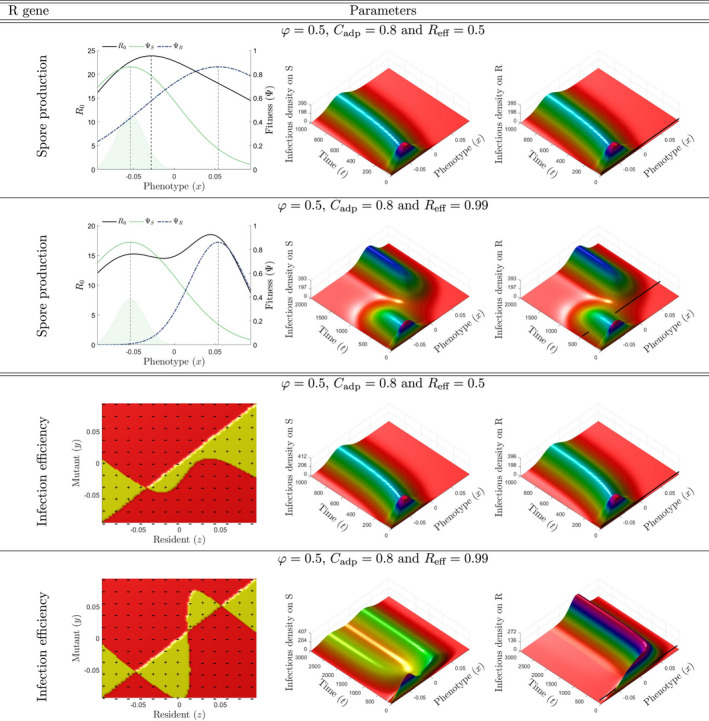
Evolutionary epidemiological dynamics. **Line 1**. The resistance impacts only spore production. The fitness function is unimodal (left panel). At t=0, the pathogen population is at its mutation–selection equilibrium on the S cultivar (light green distribution). The infection dynamics and phenotypic composition of the pathogen population on the S and R cultivars are displayed in the central and right panels, respectively. The black line in the right panel corresponds to the time to emergence *T*_emg_. **Line 2**. As in line 1, but with $R_{\text{eff}}=0.99$. The fitness function is bimodal, with both a global and a local maximum. **Line 3**. The resistance affects only infection efficiency. The pairwise invasibility plot (PIP) visualiz the sign of invasion fitness $f_{x}\left(y\right)$ (left panel). As a mutant strain y will invade the resident population *x* only if $f_{x}\left(y\right)>0$, the PIP reveals a single evolutionary attractor $\mu^{*}$ (the vertical line through $\mu^{*}$. is completely contained within a region marked “–”). **Line 4**. As in line 3 but with $R_{\text{eff}}=0.99$. The PIP reveals two evolutionary attractors (left panel). For all panels, all the other parameters are set to their reference values (Table 2). Note that the timescale axis varies between lines

In the SP scenario, the only pathogenicity trait targeted by the quantitative resistance is total spore production. As the $R_{0}$ optimization principle holds, the population always becomes monomorphic around an evolutionary attractor $\mu^{*}$ corresponding to the global maximum of the $R_{0}$ function. This happens regardless of the shape of $R_{0}$, whether $R_{0}$ has a single maximum (Figure 2, line 1) or an additional local maximum (Figure 2, line 2).

In the IE scenario, the only pathogenicity trait targeted by the quantitative resistance is infection efficiency. The optimization principle based on $R_{0}$ does not hold here, and evolutionary branching and diversification are possible and cannot be characterized by the shape of $R_{0}$. Typically, a polymorphic pathogen population can be selected at equilibrium, while $R_{0}$ has a single maximum (Figure S2). In this scenario, we need to calculate the invasion fitness to characterize the evolutionary attractors (equation 6).

**FIGURE 3:**
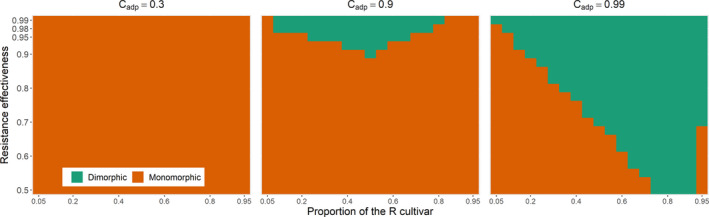
Nature of the evolutionary equilibrium (monomorphic or dimorphic) when the resistance affects only infection efficiency. The nature of the equilibrium is characterized as a function of the proportion of the R cultivar at planting (*x*‐axis) and of the relative effectiveness of the resistant cultivar (*y*‐axis) for three costs of adaptation (columns). For all panels, all the other parameters are set to their reference values (Table 2)

As in the SP scenario, the population can evolve to a monomorphic equilibrium (Figure 2, line 3). This outcome occurs for a broad range of resistance genes and deployment strategies (Figure 3). However, a dimorphic population can also be selected (Figure 2, line 4). This dimorphism is easily observed in the airborne pool of spores (Figure S3), but not necessarily on the individual cultivar (Figure 2, line 4). Indeed, the fitness functions on cultivars can give sharply different relative proportions at equilibrium (Appendix S5). A dimorphic population is selected once the cost of adaptation and resistance effectiveness reach ≥0.9 for most nonextreme proportions of the R cultivar at planting (Figure 3, central panel). Dimorphic populations are also selected in a larger number of production situations for the R cultivar characterized by the highest cost of adaptation $\left(C_{\text{adp}}=0.99\right)$. In this case, the deployment of a proportion of the R cultivar above a threshold that increases with decreasing resistance effectiveness generally leads to the selection of a dimorphic population (Figure 3, right panel).

The evolutionary epidemiology framework can also shed light on the duration of the transient dynamics. Whatever the basic resistance characteristics (pathogenicity trait targeted, resistance effectiveness, and cost of adaptation), the relative proportion of the evolutionary attractor is already ≥5% in the pool of spores in the air at the initial time point for a wide range of deployment strategies, mostly when the R cultivar is not dominant in the environment (Figure 4, lines 1 and 2, level A0). This is the case mostly when the evolutionary attractor is closer to $\mu_{S}$ (optimal phenotype on the S cultivar) than to $\mu_{R}$ (optimal phenotype on the R cultivar). In such settings, unless it already exists from the beginning in the mutation–selection equilibrium, the evolutionary attractor can be rapidly attained by mutation (Figure 2, lines 1 and 3), whether $R_{0}$ has one or two maxima (Figure 4, line 3, level "1S‐2S").

The time to emergence can substantially increase when $R_{0}$ is unimodal but with a maximum approaching the optimal phenotype on the R cultivar (Figure 4, line 3, level "1R"). Indeed, more time is then required to reach the evolutionary attractor by mutation. This increase in time, which is small with a low cost of adaptation for both scenarios (Figure 4, $C_{\text{adp}}=0.3$), is larger for higher costs (Figure 4, $C_{\text{adp}}=0.9$ or 0.99).

The longest times to emergence are obtained in the SP scenario when $R_{0}$ is bimodal with a global maximum closer to the optimal phenotype on the R cultivar. This configuration is obtained for small ranges of intermediate proportions of the R cultivar at planting combined (i) with an adaptation cost and resistance effectiveness ≥0.9, or (ii) with the highest adaptation cost considered $\left(C_{\text{adp}}=0.99\right)$ for all resistance effectiveness values tested (Figure 4, line 3, level "2R"). In this configuration, the pathogen population remains for a relatively long time around the initially dominant phenotype and then shifts by mutation to the evolutionary attractor after crossing a fitness minimum. These dynamics occur simultaneously on the S and R cultivars (Figure 2, line 2). The shape of the response surfaces for time to emergence in the IE scenario is broadly similar to that of the SP scenario. However, in all the production situations explored, time to emergence in the IE scenario was always similar to or shorter than that in the SP scenario, notably when the maximum of $R_{0}$ was closer to $\mu_{R}$. Emergence times were shorter by a mean of 41 generations when $R_{0}$ was unimodal and by a mean of 801 generations when $R_{0}$ was bimodal (Figure 4, line 3, levels "1R" and "2R"). Moreover, in the IE scenario, the evolutionary dynamics differed between cultivars (Figure 2, line 4).

### 4.3 Epidemiological outcomes

**FIGURE 5:**
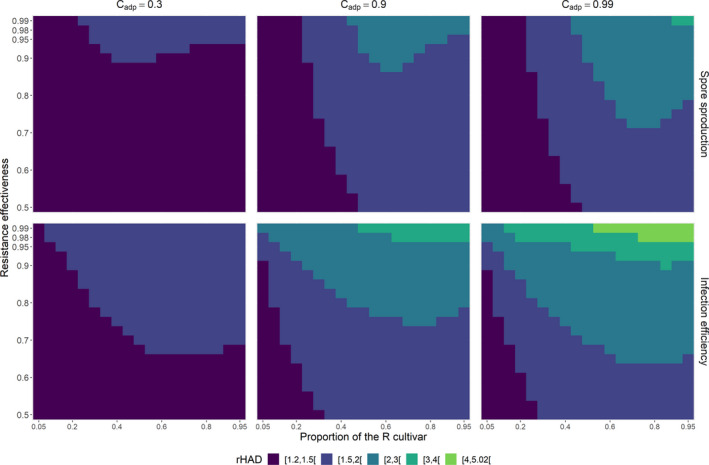
Epidemiological outcomes. Epidemiological control is estimated by calculating the gain in relative healthy area duration (rHAD), a proxy for crop yield. **Line 1**. The resistance affects only total spore production (SP scenario). Epidemiological control is characterized as a function of the proportion of the R cultivar at planting (*x*‐axis) and of the relative effectiveness of the resistant cultivar (*y*‐axis) for three costs of adaptation (columns). **Line 2**. As in line 1 when the resistance affects only infection efficiency (IE scenario). For all panels, all the other parameters are set to their reference values (Table 2)

Long‐term epidemiological control is assessed by determining the relative increase in healthy area duration. In the SP scenario, it ranges from 1.25 to 3.21, with a mean of 1.61 over the whole parameter space explored (Figure 5, line 1). Achieving a substantial relative yield gain ≥1.5 requires the deployment of an R cultivar with a cost of adaptation ≥0.9 in at least 50% of the fields, regardless of resistance effectiveness. The achievement of a higher relative yield gain ≥2 requires, in addition, a high resistance effectiveness ≥0.9.

In the IE scenario, relative yield gains range from 1.27 to 5.02, with a mean of 1.9 (Figure 5, line 1). In all the production situations explored, relative yield gains were higher for the IE scenario than for the SP scenario. The mean difference in relative yield gains between production situations was 0.28 (maximal difference 1.93). Substantial gains ≥2 are obtained for larger ranges of resistance effectiveness and proportions of the R cultivar at planting, provided that the costs of adaptation are ≥0.9. Remarkably, for both scenarios, epidemiological control is not correlated with time to emergence in the production situations explored (compare Figures 4 and 5).

## 5 DISCUSSION

This work follows a current trend toward the combined modeling of epidemiological and evolutionary dynamics in host–parasite interactions. Our theoretical framework, driven by fungal infections in plants, can be used to tackle the question of the durability of plant quantitative resistances altering specific pathogen life‐history traits. In our case study, we show that the evolutionary and epidemiological consequences of deploying resistant cultivars are not necessarily aligned, and depend on the pathogenicity trait targeted by the plant resistance genes. From an evolutionary perspective, the emergence time of the strategy best adapted to the R cultivar tends to be shorter when the resistance affects infection efficiency (IE scenario) than when it affects sporulation (SP scenario). In both cases, the emergence time is maximal for an intermediate proportion of R cultivars, as previously reported by Papaïx et al. (2018). In contrast, from an epidemiological perspective, epidemiological control is always greater in the IE scenario than in the SP scenario, as already observed by Iacono et al. (2012); Rimbaud et al. (2018). These general rules are common to all theoretical studies over the last decade investigating the epidemiological and evolutionary effects of deploying quantitative resistances in agro‐ecosystems, supporting the robustness of these conclusions.

### 5.1 Bridging the gap between modeling approaches

All these previous studies account for the interplay between epidemiological and evolutionary dynamics, but they are based on different modeling frameworks, hypotheses, and parametrizations. One of the strong points of our general modeling framework is that it allows us to bridge the gap between different modeling traditions. First, we can investigate both the short‐ and long‐term epidemiological and evolutionary dynamics of the host–pathogen interaction (Day & Proulx, 2004). The short‐term dynamics are investigated numerically, but the long‐term analysis is analytically tractable and can predict the outcome of pathogen evolution. In contrast to most studies in evolutionary epidemiology, our results are neither restricted to rare mutations, as in the classical adaptive dynamics approach, nor restricted to a Gaussian mutation kernel (see also Mirrahimi (2017)). When the trait distribution is sufficiently narrow, we expect the population to concentrate around the attractors predicted by adaptive dynamics. However, the full model is required to quantify the speed of evolution, and, notably, the time to the emergence of an adapted pathogen strain, which is often as important for practical purposes than the characterization of the potential long‐term outcome. We also note that “fat‐tailed” kernels allowing the inclusion of long‐distance dispersal events in the phenotype space could also be considered (Appendix S2).

Second, our analysis makes it possible to consider multimodal fitness functions, which are reminiscent of fitness landscapes in population genetics, and to characterize evolutionary attractors at equilibrium through a detailed description of their shapes (number of modes, steepness, and any higher moments with even order). Our results show that these features strongly affect transient dynamics, which, in turn, shape evolutionary and epidemiological outcomes. In particular, multimodal fitness functions with close local maxima result in the longest time to the emergence of adapted pathogen strains in the SP scenario. Mathematically, they are characterized by a slow convergence to equilibrium (Burie et al., 2020). In practice, it is possible to take advantage of these properties by wisely choosing the proportion of each cultivar in the agricultural landscape. Indeed, the shape of the fitness function in this scenario is basically the sum of the fitness in each hosts weighted by their proportions in the environment (equation 3).

Third, we propose a simple framework based on Gaussian distributions to describe the pathogen phenotypic landscape associated with quantitative resistance. Variation of the relative effectiveness of resistance and cost of adaptation allows us to describe a whole range of quantitative resistance effects, making it possible to consider the continuum between the two main models used for qualitative plant–parasite interactions: (i) the gene‐for‐gene model in which a parasite strain may have universal infectivity ($R_{\text{eff}}=1$ and $C_{\text{adp}}=0$) and i) the matching‐allele model in ich universal infectivity is impossible ($R_{\text{eff}}=1$ and $C_{\text{adp}}=1$) (Thrall et al., 2016). By exploring the continuum between these extremes (while noting that $R_{\text{eff}}$ and $C_{\text{adp}}$ can only tend to 1 in our framework), we can investigate in more detail the impact of deploying quantitative resistances on epidemiological and evolutionary outcomes.

### 5.2 Quantitative resistances as a major driver of pathogen evolution

The possibility that deploying R cultivars in agricultural landscapes will lead to the diversification of an initially monomorphic population and to the long‐term coexistence of different pathogen strategies has been investigated with adaptive dynamics by Gudelj, van den Bosch, et al. (2004) and by Gandon (2004) in the general context of the evolution of virulence in multihost parasites. These studies highlighted the strong dependence of evolutionary outcomes on the shape of the trade‐off curve for pathogen transmission on sympatric hosts. Concave trade‐off curves lead to monomorphic evolutionary endpoints, whereas convex or sigmoidal trade‐off curves can lead to evolutionary branching. These results can also explain the existence of sibling fungal pathogens (Gudelj, Fitt, et al. (2004)). In this approach, the transmission rate aggregates infection, spore production, and dispersal into a single proxy trait affecting secondary infection from infected to healthy hosts. In contrast, our framework allows us to disentangle the specific effect of the pathogenicity traits that follow one another during infection. Consistent with Gudelj, van den Bosch, et al. (2004), we show that dimorphism is possible with a convex or sigmoidal trade‐off for host infection efficiencies (IE scenario), whereas the population always remains monomorphic when the trade‐off curve is concave. However, our analysis shows that predictions crucially depend on the step of the parasite life cycle affected by quantitative resistance in the R cultivar. The population always remains monomorphic when resistance affects only the sporulation curve (total spore production but also any other pathogenicity traits, such as the duration of the latency period) irrespective of the underlying trade‐off curve shape (SP scenario). This is because the SP scenario allows an optimization principle based on $R_{0}$ and potential evolutionary attractors are located at the peaks of $R_{0}$ (Lion & Metz, 2018).

The evolution of plant pathogens toward generalism or specialism following the deployment of an R cultivar is also a major concern (Croll & McDonald, 2017; Papaïx et al., 2013, 2014). We propose a simple binary criterion for classifying the evolutionary attractors as generalist or specialist by comparing their $R_{0}$ on the R and S cultivars to a preset threshold (Figure S4). This highlights not only that high costs of adaptation are a major factor leading to the selection of specialists but also that emergence times and epidemiological control are weakly structured by this classification (Figure S4). It remains unclear whether it is better to favor deployment strategies leading to the selection of generalist or specialist pathogen strains, and this issue merits further investigation. The answer depends partly on the preexisting level of pathogen diversification (Papaïx, 2011). However, addressing this question in the context of quantitative resistance will first require the development of new indices measuring the degree of generalism within a specialist/generalist continuum.

### 5.3 $R_{0}$ in heterogeneous host environments sharing a common pool of propagules

Many practical epidemiological studies are based on the concept of the basic reproduction ratio $R_{0}$. For instance, van den Bosch et al. (2008) calculated $R_{0}$ for lesion‐forming foliar pathogens in a setting with two cultivars but with no effect of the time since infection on sporulation and disease‐induced mortality. $R_{0}$ is typically calculated with the spectral radius of the next‐generation operator (Diekmann et al., 1990), but we follow here a methodology based on the generation evolution operator (Inaba, 2012) to derive an expression for the basic reproduction number $R_{0}$ in heterogeneous host populations with any number of cultivars. This expression captures the time scales inherent to the life cycle of plant fungal pathogens with, notably, sporulation varying over time. Capturing such patterns is a challenge in the modeling of plant diseases, as reported by Cunniffe et al. (2015). Moreover, with a common pool of well‐mixed airborne pathogen propagules, the function $R_{0}\left(x\right)$ is an exact fitness proxy for competing strains with potentially different sporulation curves (including the latent period, total spore production, and shape parameters). This is important because the computation of $R_{0}\left(x\right)$ usually assumes that the invading pathogen enters an uninfected host population. However, more generally, a clear distinction between pathogen invasion fitness $R\left(x,y\right)$ and epidemiological $R_{0}\left(x\right)$ is required for correct discussions of the adaptive evolution of pathogens (Lion & Metz, 2018). Even with a common pool of spores, the optimization principle of $R_{0}\left(x\right)$ does not hold when infection efficiencies differ between host classes.

### 5.4 Notes on model assumptions

The model assumes an infinitely large pathogen population. Demographic stochasticity is, thus, ignored despite its potential impact on evolutionary dynamics (e.g., lower probabilities of emergence and fixation of beneficial mutations, and reduction of standing genetic variation (Kimura, 1962)). In particular, genetic drift is more likely to affect the maintenance of a neutral polymorphism than that of a protected polymorphism when selection favors the coexistence of different genotypes protecting against invasions by mutant strategies (Geritz et al., 1998). The effect of genetic drift depends on the stability properties of the model considered. As our model has a unique globally stable eco‐evolutionary equilibrium, genetic drift is likely to have a weaker impact than in models with several locally stable equilibria. Moreover, the large effective population sizes (from 10^3^ to 3.10^4^) reported at field scale for several species of wind‐dispersed, spore‐producing plant pathogens (Ali et al., 2016; Walker et al., 2017; Zhan et al., 2001) suggest a weak effect of genetic drift.

The model assumes that the aggressiveness components are mutually independent. However, correlations between traits have sometimes been reported. For instance, Pariaud et al. (2013) observed a positive correlation between the duration of the latent period and the fecundity of a plant fungus. This relationship describes a phenotypic trade‐off because a short latent period and a high sporulation probability represent fitness advantages. It can be introduced into the SP scenario assuming that the latent period and the total spore production of a pathogen strain *x* are linked by mathematical functions, such as the quadratic relationship suggested by Pariaud et al. (2013). Alternatively, correlations between pathogen life‐history traits can emerge from the covariance matrix of the (multidimensional) mutation kernel (Gandon, 2004). Another feature of the framework that we did not use is the possibility of tracking the evolution of the quantitative traits of the pathogen with Price's equation (Day & Gandon, 2007; Day & Proulx, 2004; Iacono et al., 2012). Indeed, differential equations for the mean phenotype and phenotypic variance of any trait of interest can be derived from model (2).

The model also assumes a unique pool of well‐mixed propagules. Spore dispersal therefore disregards the location of healthy and infected hosts. This assumption, which ensures the one‐dimensional environmental feedback loop of the model, is more likely when the extent of the field or landscape considered is not overly large with respect to the dispersal function of airborne propagules. Airborne fungal spores often disperse over substantial distances. Mean dispersal distances range from 100 m to 1 km and, in most cases, long‐distance dispersal events are frequent (Fabre et al., 2021). A spatially implicit framework embedded into integro‐differential equations was recently used to describe the eco‐evolutionary dynamics of a phenotypically structured population subject to mutation, selection, and migration between two habitats (Mirrahimi, 2017). With the same goal, Rimbaud et al. (2018) used a spatially explicit framework with several habitats embedded in an HEIR stochastic model. It would be interesting to draw on these examples and extend our approach to a spatially explicit environment. Indeed, when dispersal decreases with distance, large homogeneous habitats promote diversification, whereas smaller habitats, favoring migration between different patches, hamper diversification (Débarre & Gandon, 2010; Haller et al., 2013; Papaïx et al., 2013, 2014).

### 5.5 Applied significance

This study gave rise to three key applied findings. The first is the $R_{0}$ equation in an environment with several cultivars. This equation is based on a gamma sporulation curve, as documented for several plant fungi (van den Bosch et al., 1988; van den Bosch et al., 1988; Kolnaar & Bosch, 2001; Sache et al., 1997; Segarra et al., 2001), and explicitly integrates the pathogenicity traits expressed during the basic steps of infection (infection efficiency, latent period, sporulation dynamics). As these traits can be measured in the laboratory, this expression of $R_{0}$ bridges the gap between plant‐scale and epidemiological studies, and between experimental and theoretical approaches. It helps address the practical difficulty of estimating $R_{0}$ for real populations, as pointed out by Gilligan and van den Bosch (2008). For example, it can be used to compare the fitness of a collection of pathogen isolates (e.g., see Montarry et al. (2010) for an application to potato late blight) or to predict their field structure (e.g., see Durand et al. (2017) for an application to Lyme disease).

The second applied finding is that the choice of quantitative resistance genes operated by plant breeders drives pathogen diversification. This effect is mediated by the parasite life cycle targeted by the resistance. Quantitative resistances specifically affecting different stages of pathogen cycles have been identified in several pathosystems (e.g., Azzimonti et al. (2013), Chung et al. (2010), Jorge et al. (2005)). Moreover, understanding the conditions for maintaining pathogen polymorphism is a long‐standing question in disease ecology and evolution, with implications for disease management (Vale, 2013). We show here that the pathogen population always remains monomorphic when resistance affects only sporulation, irrespective of the underlying trade‐off curve shape. In contrast, pathogen diversification is possible when resistance affects only infection efficiency. This finding highlights the need for a detailed knowledge of the effect of resistance genes on pathogen life cycle for a full understanding of the impact of the deployment of quantitative resistances on the strain structures underlying the patterns of disease incidence. This finding is important, because quantitative resistances are increasingly being used by plant breeders to deal with the rapidity with which major resistance genes break down (Zhan et al., 2015). Interestingly, similar questions concerning the epidemiological and evolutionary consequences of vaccination strategies have been considered. Quantitative resistance traits targeting pathogen infection rate, latent period, and sporulation rate are analogous to partially effective (imperfect) vaccines with anti‐infection, antigrowth, and antitransmission modes of action, respectively (Gandon et al., 2001). Similarly, the proportion of the R cultivar deployed is analogous to vaccination coverage in the population; in the vaccination context, the relative effectiveness of resistance is termed "vaccine efficacy" and the relative cost of adaptation is termed "cost of escape" (Gandon & Day, 2007).

Finally, using a different theoretical approach, we confirm the finding of Papaïx et al. (2018) that "there is no silver bullet deployment strategy" simultaneously maximizing epidemiological and evolutionary outcomes (Rimbaud et al., 2021). Accordingly, as the different stakeholders involved in plant resistance management may pursue objectives that are not always compatible (e.g., growers, breeders), they should all be involved in defining the best management strategies.

## AUTHORS’ CONTRIBUTIONS

RDD, AD, JBB, and FF planned and designed the research. RDD, AD, JBB, and FF developed analytic tools and conducted numerical experiments. SL and QR critically revised the manuscript and contributed to its writing. All the authors gave final approval for publication and agreed to be held accountable for the work described.

## Supporting information

Supplementary Material

## Data Availability

The MATLAB codes used to simulate the model and generate the main figures have been deposited in Dataverse at https://doi.org/10.15454/WAEIMA
