# Supplementary material for: An epi‐evolutionary model for predicting the adaptation of spore‐producing pathogens to quantitative resistance in heterogeneous environments
Source: Evol Appl. 2021 Dec 31;15(1):95–110. doi: 10.1111/eva.13328 (PMC8792485; doi:10.1111/eva.13328)
Supplement: Supplementary file 1 — Supplementary Material [file EVA-15-95-s001.pdf]

An epi-evolutionary model for predicting the adaptation of spore-producing pathogens to quantitative resistance in heterogeneous environments

Frédéric Fabre[a], Jean-Baptiste Burie[b,c], Arnaud Ducrot[d], Sébastien Lion[e], Quentin Richard[f], Ramsès Djidjou-Demasse[f]

[a] *INRAE, Bordeaux Sciences Agro, UMR SAVE, Villenave d'Ornon F-33882, France*

[b] *CNRS, IMB, UMR 5251, Talence F-33400, France.*

[c] *Univ. Bordeaux, IMB, UMR 5251, Talence F-33400, France*

[d] *Univ. Normandie, UNIHAVRE, LMAH, FR-CNRS-3335, ISCN, 76600 Le Havre, France*

[e] *CEFE, CNRS, Univ. Montpellier, Univ. Montpellier 3 Paul-Valéry, EPHE, IRD, Montpellier F-34293, France*

[f] *MIVEGEC, Univ. Montpellier, IRD, CNRS, Montpellier, France*

December 18, 2021

This file provides the supporting information of https://doi.org/10.1111/eva.13328

Abstract

We have modeled the evolutionary epidemiology of spore-producing plant pathogens in heterogeneous environments sown with several cultivars carrying quantitative resistances. The model explicitly tracks the infection-age structure and genetic composition of the pathogen population. Each strain is characterized by pathogenicity traits determining its infection efficiency and a time-varying sporulation curve taking into account lesion aging. We first derived a general expression of the basic reproduction number $\mathcal{R}_0$ for fungal pathogens in heterogeneous environments. We show that the evolutionary attractors of the model coincide with local maxima of $\mathcal{R}_0$ only if the infection efficiency is the same on all host types. We then studied the contribution of three basic resistance characteristics (the pathogenicity trait targeted, resistance effectiveness, and adaptation cost), in interaction with the deployment strategy (proportion of fields sown with a resistant cultivar) to (i) pathogen diversification at equilibrium and (ii) the shaping of transient dynamics from evolutionary and epidemiological perspectives. We show that quantitative resistance affecting only the sporulation curve will always lead to a monomorphic population, whereas dimorphism (*i.e.* pathogen diversification) can occur if resistance alters infection efficiency, notably with high adaptation costs and proportions of the resistant cultivar. Accordingly, the choice of the quantitative resistance genes operated by plant breeders is a driver of pathogen diversification. From an evolutionary perspective, the time to emergence of the evolutionary attractor best adapted to the resistant cultivar tends to be shorter when resistance affects infection efficiency than when it affects sporulation. Conversely, from an epidemiological perspective, epidemiological control is always greater when the resistance affects infection efficiency. This highlights the difficulty of defining deployment strategies for quantitative resistance simultaneously maximizing epidemiological and evolutionary outcomes.

Key words. Basic reproduction number; Resistance durability; Adaptive dynamics; Spore-producing pathogens; Quantitative resistance; Integro-differential equations.

A Supplementary figure

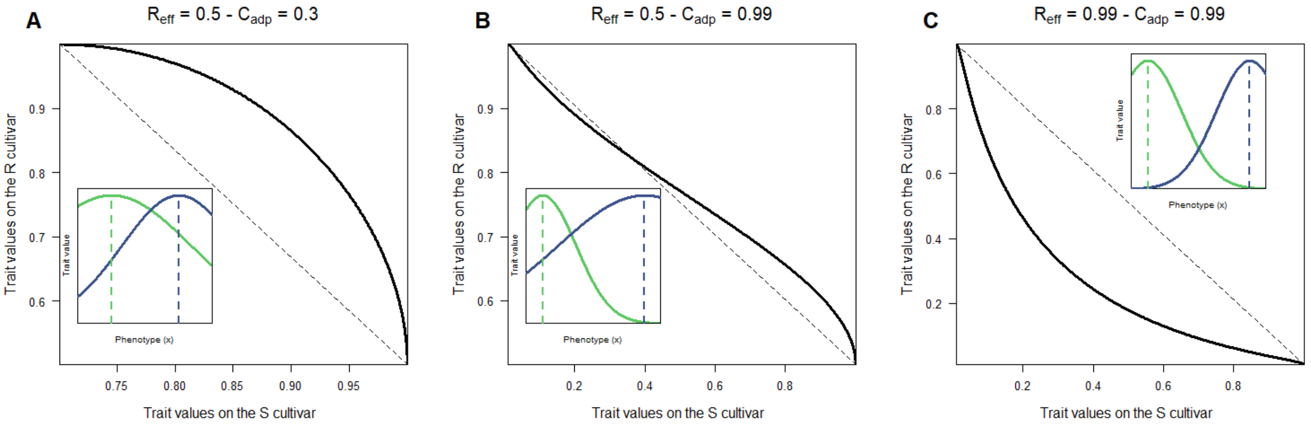

Figure S1: Shapes of the trade-off curves of the pathogen traits values on the S cultivar against those on the R cultivar as given by $\omega_R = f(\omega_S)$ with $x \in [\mu_S, \mu_R]$. The trade-off can be either concave (panel A), sigmoidal (panel B) or convexe (panel C). Inset graphs show the corresponding Gaussian functions $\omega_S(x)$ and $\omega_R(x)$ as in Figure 1B. For all panels all other parameters are set to their reference values (Table 2).

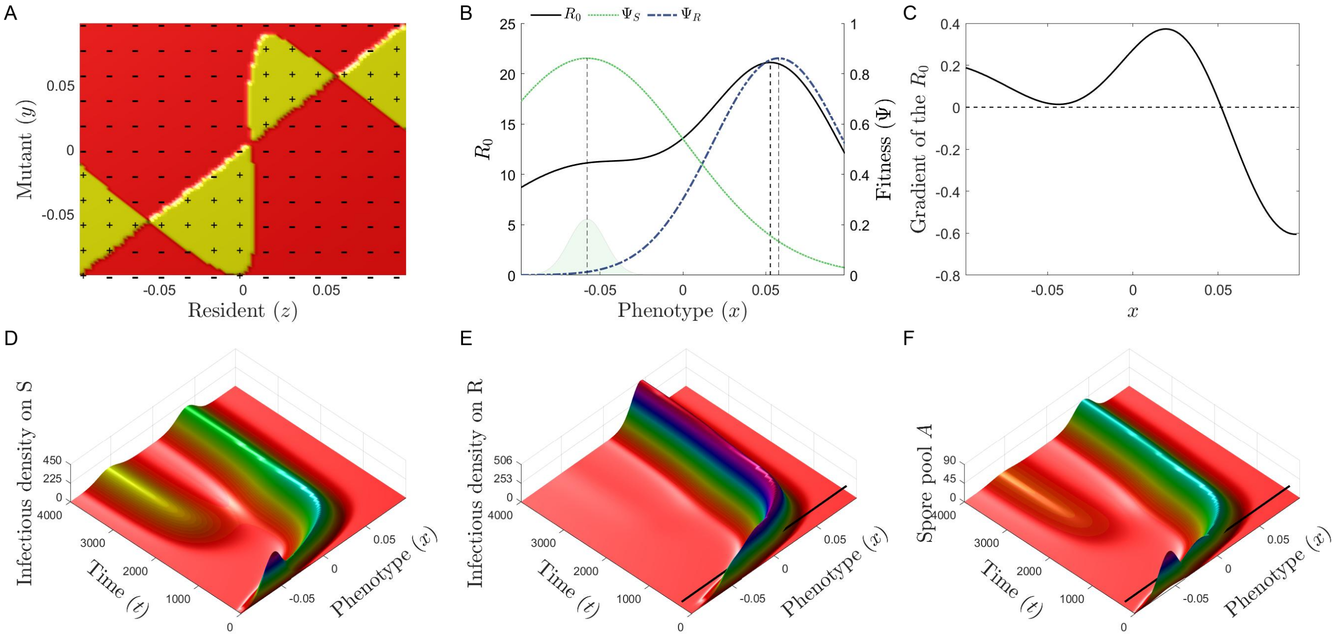

Figure S2: An example of configuration where a polymorphic pathogen population is selected at equilibrium (panels A, D, E, F) while a single local maximum exists for $\mathcal{R}_0$ (panel B) as confirmed by its gradient (panel C). Parameters values are $\varphi = 0.64$, $\sigma_S = 0.06$, $C_{\text{adp}} = 0.845$ and $R_{\text{eff}} = 0.985$ and $\varepsilon = 0.002$. Other parameters are set to their reference values. The PIP in panel A visualizes the sign of the invasion fitness $f_x(y)$. The mutant strain y will invade the resident population x if and only if $f_x(y) > 0$.

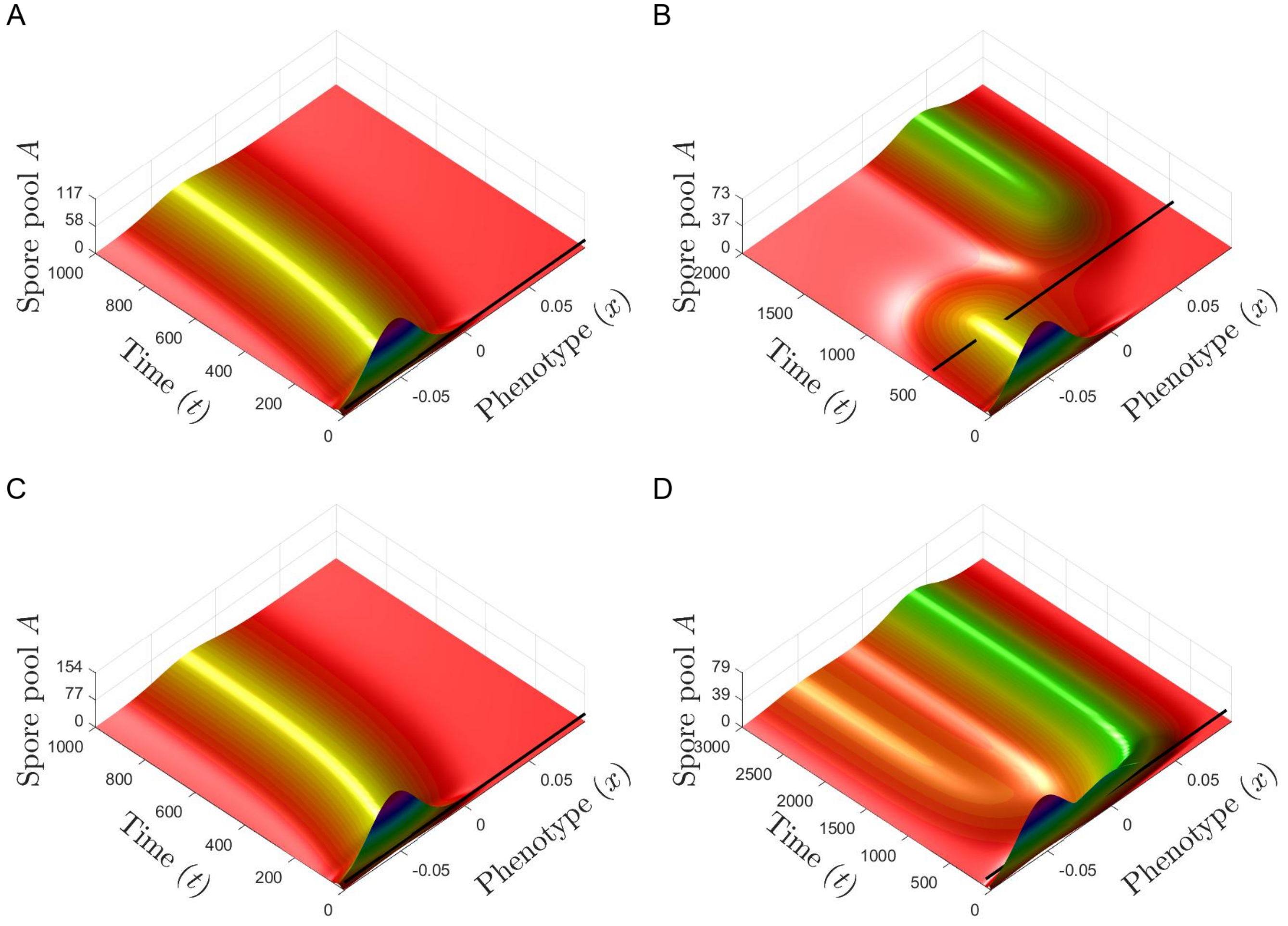

Figure S3: Dynamics of the density of airborne pool of pathogen $A(t,x)$. **A-B**. The resistance impacts the total spore production (SP scenario). The dynamics of $A(t,x)$ corresponds to the lines 1 and 2 of Figure 2: (A) $\varphi = 0.5$, $C_{\text{adp}} = 0.8$ and $R_{\text{eff}} = 0.5$ and (B) $\varphi = 0.5$, $C_{\text{adp}} = 0.8$ and $R_{\text{eff}} = 0.99$. **C-D**. The resistance impacts the pathogen infection efficiency (IE scenario). The dynamics of $A(t,x)$ corresponds to the lines 3 and 4 of Figure 2: (C) $\varphi = 0.5$, $C_{\text{adp}} = 0.8$ and $R_{\text{eff}} = 0.5$ and (D) $\varphi = 0.5$, $C_{\text{adp}} = 0.8$ and $R_{\text{eff}} = 0.99$. For all panels, all other parameters are set to their reference values (Table 2).

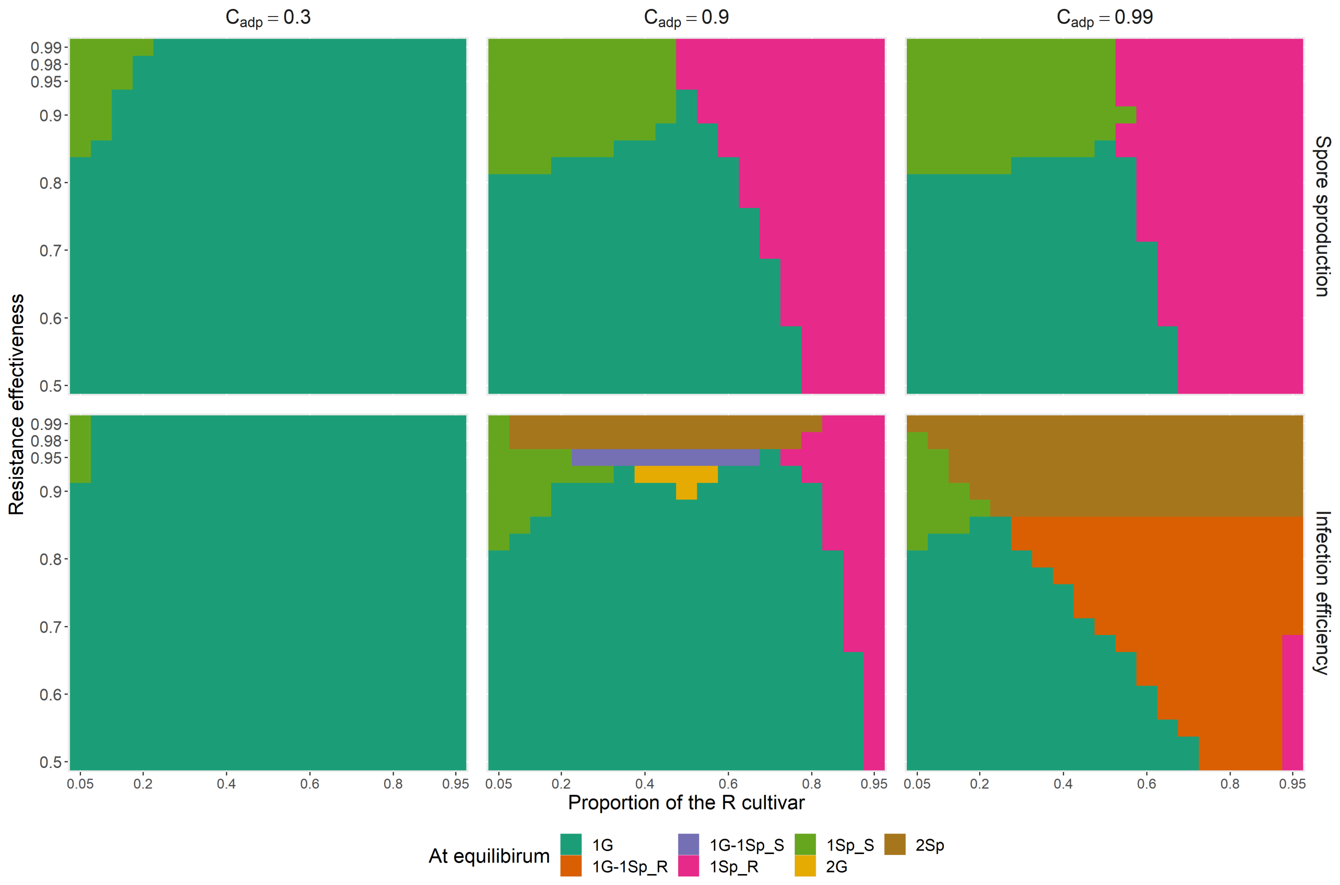

Figure S4: Characterization as generalist and/or specialist of the evolutionary attractors. Due to the quantitative interactions considered, a phenotype μ^* has never strictly a single host range, and its characterization depends on a threshold set here to 0.2. Accordingly, μ^* is termed a specialist of the R cultivar if $\mathcal{R}_0^S(\mu^*)/\mathcal{R}_0^R(\mu^*) < 0.2$, a specialist of the S cultivar if $\mathcal{R}_0^R(\mu^*)/\mathcal{R}_0^S(\mu^*) < 0.2$, and a generalist otherwise. **Line 1**. The resistance impacts only the total spore production (SP scenario). The evolutionary attractor is characterized as a function of the proportion of the R cultivar at planting (x-axis) and of the relative effectiveness of the resistant cultivar (y-axis) for three costs of adaptation (columns). The equilibrium is always monomorphic in the SP scenario with one generalist (1 G), one specialist of the S cultivar (1 SpS) or one specialist of the R cultivar (1 SpR). **Line 2**. The resistance impacts only the pathogen infection efficiency (IE scenario). In the IE scenario, the equilibrium can also be dimorphic with two specialists (2 Sp), one generalist and one specialist of the S cultivar (1G & 1 SpS), one generalist and one specialist of the R cultivar (1G & 1 SpR) or two generalists (2 G). For all panels, all other parameters are set to their reference values (Table 2).

B Properties of the mutation kernel m_ε

In the model, mutations randomly displace strains into the phenotype space at each generation according to the kernel m_ε. In the simulations, we used a centered multivariate Gaussian distribution with standard deviation ε. It leads to $m_\varepsilon(x-y) = \frac{1}{\varepsilon^N} m\left(\frac{x-y}{\varepsilon}\right)$. However, the kernel is not restricted to Gaussian distributions. For example, any exponential-power kernels are possible and, in particular, "fat-tailed" exponential-power kernels which allow long-distance dispersal events into the phenotype space can be considered (Klein et *al.*, 2006).

More generally, the kernel function m_ε arising in model (2) should satisfy the following properties:

(H1) The function m_ε is almost everywhere strictly positive on $\mathbb{R}^N$ and should be normalised such that,

$$\int_{\mathbb{R}^N} m_\varepsilon(x)\mathrm{d}x = 1.$$

This last condition expresses that all interactions generated on the phenotypic space of pathogens necessarily end up somewhere on that space.

(H2) Its variation should only depend on the distance separating the points between which the interactions are evaluated (*i.e.* $m_\varepsilon(x) = m_\varepsilon(-x)$, for all $x \in \mathbb{R}^N$).

(H3) It decays rather fast at infinity in the sense that $m_\varepsilon(x) = \varepsilon^{-N} m(x/\varepsilon)$ and $m(x) = O\left(\frac{1}{\|x\|^\infty}\right)$ as $\|x\| \to \infty$. In other words, $\lim_{|x|\to\infty} |x|^n m(x) = 0$, for all $n \in \mathbb{N}$. This assumption does not mean the mutation kernel has a very fast decay at infinity. We emphasize that the decay of the mutation kernel distribution considered here allows considering the tails of a wide variety of distributions.

References

Klein, E.K., Lavigne, C., Gouyon, P.H. (2006). Mixing of propagules from discrete sources at long distance: comparing a dispersal tail to an exponential. *BMC Ecology*, 6, 3.

C Some special cases of the general model (2)

By omitting the age structure, we re-write model (2) as follows

$$\begin{cases} \dfrac{\partial}{\partial t} H_k(t) = \varphi_k \Lambda - \theta H_k(t) - H_k(t) \displaystyle\int_{\mathbb{R}^N} \beta_k(y) A_k(t,y)\mathrm{d}y, \\ \dfrac{\partial}{\partial t} I_k(t,x) = \beta_k(x) H_k(t) A(t,x) - \left(\theta + d_k(x) + \dfrac{1}{l_k(x)}\right) I_k(t,x), \\ \dfrac{\partial}{\partial t} A(t,x) = -\delta A(t,x) + \displaystyle\sum_{k=1}^{N_c} \int_{\mathbb{R}^N} m_\varepsilon(y-x) p_k(y) I_k(t,y)\mathrm{d}y, \end{cases} \tag{C.1}$$

wherein we take into account the (host and strain-specific) duration of the sporulation period, denoted by $l_k(x)$.

Furthermore, if we assume that there are no "interactions" in the phenotypic space of pathogens, *i.e.* without mutations: $\varepsilon \to 0$, then the simplified model (C.1) rewrites

$$\begin{cases} \dfrac{\partial}{\partial t} H_k(t) = \varphi_k \Lambda - \theta H_k(t) - H_k(t) \displaystyle\int_{\mathbb{R}^N} \beta_k(y) A_k(t,y)\mathrm{d}y, \\ \dfrac{\partial}{\partial t} I_k(t,x) = \beta_k(x) H_k(t) A(t,x) - \left(\theta + d_k(x) + \dfrac{1}{l_k(x)}\right) I_k(t,x), \\ \dfrac{\partial}{\partial t} A(t,x) = \displaystyle\sum_{k=1}^{N_c} p_k(x) I_k(t,x) - \delta A(t,x). \end{cases} \tag{C.2}$$

D The fitness function

In this appendix we explain how to compute the fitness function. To that aim, by formally taking the limit $\varepsilon \to 0$ into model (2), this system becomes

$$\begin{cases} \dfrac{\partial}{\partial t} H_k(t) = \varphi_k \Lambda - \theta H_k(t) - H_k(t) \displaystyle\int_{\mathbb{R}^N} \beta_k(y) A(t,y)\mathrm{d}y, \\ \left(\dfrac{\partial}{\partial t} + \dfrac{\partial}{\partial a}\right) I_k(t,a,x) = -\left(\theta + d_k(a,x)\right) I_k(t,a,x), \\ I_k(t,0,x) = \beta_k(x) H_k(t) A(t,x), \\ \dfrac{\partial}{\partial t} A(t,x) = \displaystyle\sum_{k=1}^{N_c} \int_0^\infty r_k(a,x) I_k(t,a,x)\mathrm{d}a - \delta A(t,x). \end{cases} \tag{D.3}$$

Let us assume that system (D.3) reaches a monomorphic epidemiological equilibrium $E^z = (H_k^z, I_k^z(\cdot)\delta_z, A^z\delta_z)_{k=1,\cdots N_c}$, for some trait z, before a new mutation with trait value, say, y occurs. Note that E^z is the environmental feedback of the resident z. We introduce a small perturbation in (D.3) in the phenotype trait y, so that the evolution of the system reads as follows: $H_k(t) = H_k^z + u_k(t)$ and

$$I_k(t,a,x) = I_k^z(a)\delta_z(x) + j_k(t,a)\delta_y(x) \text{ and } A(t,x) = A^z\delta_z(x) + B(t)\delta_y(x),$$

and the small perturbations for the infection, j_k and B, are governed by the linearized system of equations around E^z. This reads as

$$\begin{cases} \left(\dfrac{\partial}{\partial t} + \dfrac{\partial}{\partial a}\right) j_k(t,a) = -\left(\theta + d_k(a,y)\right) j_k(t,a), \\ j_k(t,0) = \beta_k(y) H_k^z B(t), \\ B'(t) = \displaystyle\sum_{k=1}^{N_c} \int_0^\infty r_k(a,y) j_k(t,a)\mathrm{d}a - \delta B(t). \end{cases} \tag{D.4}$$

In order to study the evolution of this perturbation we will derive a renewal equation on $b^z(t,y)$, the density of newly produced spores at time t with phenotype y in the resident population with phenotype x. This term is more precisely defined by

$$b^z(t,y) = \sum_{k=1}^{N_c} \int_0^\infty r_k(a,y) j_k(t,a)\mathrm{d}a.$$

It then follows from the j_k-equation of the linear system (D.4), that

$$j_k(t,a) = \begin{cases} j_k(0,a-t) e^{-\theta t - \int_{a-t}^a d_k(\sigma,y)\mathrm{d}\sigma}, & a \geq t, \\ \beta_k(y) H_k^z B(t-a) e^{-\theta a - \int_0^a d_k(\sigma,y)\mathrm{d}\sigma}, & a < t, \end{cases}$$

while

$$B(t) = \int_0^t b^z(s,y) e^{-\delta(t-s)}\mathrm{d}s + B(0)e^{-\delta t}.$$

As a consequence, $b^z(t,y)$ satisfies the following renewal equation:

$$b^z(t,y) = \sum_{k=1}^{N_c} H_k^z \int_0^t r_k(a,y)\beta_k(y) \int_0^{t-a} b^z(s,y) e^{-\delta(t-a-s)} e^{-\theta a - \int_0^a d_k(\sigma,y)\mathrm{d}\sigma}\mathrm{d}s\mathrm{d}a + \mathcal{F}(t,y,z), \tag{D.5}$$

wherein we have set

$$\begin{aligned} \mathcal{F}(t,y,z) = & \sum_{k=1}^{N_c} \int_t^\infty r_k(a,y) j_k(0,a-t) e^{-\theta t - \int_{a-t}^a d_k(\sigma,y)\mathrm{d}\sigma}\mathrm{d}a \\ & + B(0) \sum_{k=1}^{N_c} H_k^z \int_0^t r_k(a,y)\beta_k(y) e^{-\delta(t-a)} e^{-\theta a - \int_0^a d_k(\sigma,y)\mathrm{d}\sigma}\mathrm{d}a. \end{aligned}$$

Then (D.5) can be rewritten as

$$b^z(t,y) = \int_0^t B^z(a,y) b^z(t-a,y)\mathrm{d}a + \mathcal{F}(t,y,z),$$

where $B^z(a,y)$ is the expected number of new infections produced per unit time, in a resident host population with phenotype z, by an individual which was infected a units of time ago with the phenotype y, given by

$$B^z(a,y) = e^{-\delta a} \sum_{k=1}^{N_c} H_k^z \beta_k(y) \int_0^a r_k(s,y) e^{\delta s - \theta s - \int_0^s d_k(\sigma,y)\mathrm{d}\sigma} \mathrm{d}s.$$

Due to the above formulation, it follows from classical adaptive dynamics (Diekmann et *al.*, 2005; Geritz et *al.*, 1997; Metz et *al.*, 1996) that the spore numbers, $\mathcal{R}(y, E^z)$, of a rare mutant strategy, y, in the resident z-population is given by

$$\mathcal{R}(y, E^z) = \int_0^\infty B^z(a,y)\mathrm{d}a = \sum_{k=1}^{N_c} H_k^z \Psi_k(y),$$

wherein $\Psi_k(y) = \frac{1}{\delta}\beta_k(y) \int_0^\infty r_k(a,y) \exp\left(-\theta a - \int_0^a d_k(\sigma,y)\mathrm{d}\sigma\right) \mathrm{d}a$. Then, the invasion fitness $f_z(y)$ of a mutant strategy y in the resident z-population is given by

$$f_z(y) = \mathcal{R}(y, E^z) - 1 = \sum_{k=1}^{N_c} H_k^z \Psi_k(y) - 1. \tag{D.6}$$

Note that when the environmental feedback E^z is reduced to the disease-free environment, then S_k^z re-writes as $S_k^z = \frac{\Lambda \varphi_k}{\theta}$. And the epidemiological basic reproduction number of the pathogen with the phenotype y is calculated as

$$\mathcal{R}_0(y) = \frac{\Lambda}{\theta}\Psi(y), \quad \text{with} \quad \Psi = \sum_{k=1}^{N_c} \varphi_k \Psi_k.$$

Once the pathogen has spread and reached the monomorphic equilibrium, the endemic feedback environment E^z becomes

$$H_k^z = \frac{\Lambda \varphi_k}{\theta + \beta_k(z) A^z}, \quad I_k^z(a) = \beta_k(z) A^z H_k^z \exp\left(-\theta a - \int_0^a d_k(\sigma, z)\mathrm{d}\sigma\right), \tag{D.7}$$

where $A^z > 0$ is the unique solution of the following equation (only defined when $\mathcal{R}_0(z) > 1$):

$$\sum_{k=1}^{N_c} \frac{\Lambda \varphi_k}{\theta + \beta_k(z) A^z} \Psi_k(z) = 1. \tag{D.8}$$

References

Diekmann, O., Jabin, P.E., Mischler, S., Perthame, B. (2005). The dynamics of adaptation: an illuminating example and a Hamilton-Jacobi approach. *Theoretical Population Biology*, 67, 257-271.

Geritz, S.A., Metz, J.A., Kisdi, É., Meszéna, G. (1997). Dynamics of adaptation and evolutionary branching. *Physical Review Letters*, 78(10), 2024-2027.

Metz, J.A.J., Geritz, S.A.H., Meszéna, G., Jacobs, F.J.A., van Heerwaarden, J.S. (1996). Adaptive dynamics, a geometrical study of the consequences of nearly faithful reproduction. Pages 183-231 in: Stochastic and spatial Structures of Dynamical Systems. van Strien, S.J., Verduyn Lunel, S.M., eds. North-Holland, Amsterdam.

E Dimorphic or monomorphic equilibrium

To simplify the presentation, we consider system (2) with $N_c = 2$ corresponding to S and R cultivars. Denote by $(H_0, i_0(\cdot), A_0)$ the endemic equilibrium of system (2) as $\varepsilon \to 0$ and when only S is cultivated (*i.e.* when the proportion φ of R is zero). From results in Djidjou-Demasse et *al.* (2017) we have

$$H_0 = \frac{1}{\Psi_S(\mu_S)}. \tag{E.9}$$

Now, let $(H_S, H_R, i_S(\cdot), i_R(\cdot), A)$ be an equilibrium of system (2) when a proportion $\varphi > 0$ of R is cultivated. Next recall that, for $k \in \{S, R\}$,

$$H_k = \frac{\varphi_k \Lambda}{\theta + \int_{\mathbb{R}^N} \beta_k(z) A(z) \mathrm{d}z} \text{ and } I_k(x,a) = \beta_k(x) A(x) H_k \exp\left(-\theta a - \int_0^a d_k(\sigma, x) \mathrm{d}\sigma\right),$$

so that $A(\cdot)$ becomes a solution of the nonlinear equation:

$$\int_{\mathbb{R}^N} m_\varepsilon(y-x) \sum_{k\in\{S,R\}} \frac{\varphi_k \Psi_k(y)}{\theta + \int_{\mathbb{R}^N} \beta_k(z) A(z)\mathrm{d}z} A(y) \mathrm{d}y = \frac{1}{\Lambda} A(x). \tag{E.10}$$

Using this equation we heuristically explore conditions yielding to dimorphic or monomorphic equilibrium.

Quantitative resistance impacting infection efficiencies β_S and β_R (IE scenario). We formally assume that the population of spores writes $A(x) = a_S \delta_{\mu_S}(x) + a_R \delta_{\mu_R}(x)$, and we plug this ansatz into equation (E.10) above. This yields, for any x,

$$\begin{aligned} & a_R \left[\frac{\varphi \Psi_R(\mu_R)}{\theta + a_R \beta_R(\mu_R) + a_S \beta_R(\mu_S)} + \frac{(1-\varphi)\Psi_S(\mu_R)}{\theta + a_R\beta_S(\mu_R) + a_S\beta_S(\mu_S)}\right] m_\varepsilon(\mu_R - x) \\ & + a_S \left[\frac{\varphi \Psi_R(\mu_S)}{\theta + a_R \beta_R(\mu_R) + a_S \beta_R(\mu_S)} + \frac{(1-\varphi)\Psi_S(\mu_S)}{\theta + a_R\beta_S(\mu_R) + a_S\beta_S(\mu_S)}\right] m_\varepsilon(\mu_S - x) \\ & = \frac{1}{\Lambda}\left[a_R \delta_{\mu_R}(x) + a_S \delta_{\mu_S}(x)\right]. \end{aligned}$$

Letting $\varepsilon \to 0$ and recalling that $m_\varepsilon(x) \approx \delta_0(x)$, one obtains

$$\begin{aligned} & a_R \left[\frac{\varphi \Psi_R(\mu_R)}{\theta + a_R \beta_R(\mu_R) + a_S \beta_R(\mu_S)} + \frac{(1-\varphi)\Psi_S(\mu_R)}{\theta + a_R\beta_S(\mu_R) + a_S\beta_S(\mu_S)}\right] \delta_{\mu_R}(x) \\ & + a_S \left[\frac{\varphi \Psi_R(\mu_S)}{\theta + a_R \beta_R(\mu_R) + a_S \beta_R(\mu_S)} + \frac{(1-\varphi)\Psi_S(\mu_S)}{\theta + a_R\beta_S(\mu_R) + a_S\beta_S(\mu_S)}\right] \delta_{\mu_S}(x) \\ & = \frac{1}{\Lambda}\left[a_R \delta_{\mu_R}(x) + a_S \delta_{\mu_S}(x)\right], \end{aligned}$$

that is

$$\begin{cases} \dfrac{a_R \varphi \Psi_R(\mu_R)}{\theta + a_R \beta_R(\mu_R) + a_S \beta_R(\mu_S)} + \dfrac{a_R(1-\varphi)\Psi_S(\mu_R)}{\theta + a_R\beta_S(\mu_R) + a_S\beta_S(\mu_S)} = \dfrac{a_R}{\Lambda}, \\ \dfrac{a_S \varphi \Psi_R(\mu_S)}{\theta + a_R \beta_R(\mu_R) + a_S \beta_R(\mu_S)} + \dfrac{a_S(1-\varphi)\Psi_S(\mu_S)}{\theta + a_R\beta_S(\mu_R) + a_S\beta_S(\mu_S)} = \dfrac{a_S}{\Lambda}. \end{cases}$$

As a consequence, for the equilibrium to be dimorphic, namely $a_R > 0$ and $a_S > 0$, it is necessary that there exist $a_R > 0$ and $a_S > 0$ satisfying the following system of equations:

$$\begin{cases} \dfrac{\varphi \Psi_R(\mu_R)}{\theta + a_R \beta_R(\mu_R) + a_S \beta_R(\mu_S)} + \dfrac{(1-\varphi)\Psi_S(\mu_R)}{\theta + a_R\beta_S(\mu_R) + a_S\beta_S(\mu_S)} = \dfrac{1}{\Lambda}, \\ \dfrac{\varphi \Psi_R(\mu_S)}{\theta + a_R \beta_R(\mu_R) + a_S \beta_R(\mu_S)} + \dfrac{(1-\varphi)\Psi_S(\mu_S)}{\theta + a_R\beta_S(\mu_R) + a_S\beta_S(\mu_S)} = \dfrac{1}{\Lambda}. \end{cases}$$

We set

$$\begin{cases} X = X(a_S, a_R) = \left(\theta + a_R\beta_R(\mu_R) + a_S\beta_R(\mu_S)\right)^{-1}, \\ Y = Y(a_S, a_R) = \left(\theta + a_R\beta_S(\mu_R) + a_S\beta_S(\mu_S)\right)^{-1}. \end{cases} \tag{E.11}$$

Recall that with the IE scenario we have $r_S = r_R$ and $d_S = d_R$ such that the fitness function

$$\Psi_k(x) = \frac{1}{\delta}\beta_k(x) \int_0^\infty r_k(a,x) \exp\left(-\theta a - \int_0^a d_k(\sigma,x)\mathrm{d}\sigma\right) \mathrm{d}a,$$

takes the form $\Psi_k = c_0 \beta_k$ for $k = S, R$ (where c_0 is the same positive funtional for S and R). Doing that, the above system rewrites

$$\mathcal{K}(X,Y)^T = \frac{1}{c_0 \Lambda}(1,1)^T, \tag{E.12}$$

wherein $\mathcal{K} = \begin{pmatrix} \varphi\beta_R(\mu_R) & (1-\varphi)\beta_S(\mu_R) \\ \varphi\beta_R(\mu_S) & (1-\varphi)\beta_S(\mu_S) \end{pmatrix}$ and $c_0 = c_0(\mu_S) = c_0(\mu_R)$. With the IE scenario (*i.e.* with trade-off on infection efficiency β_k), we reasonably have $\beta_R(\mu_R) > \beta_R(\mu_S)$ and $\beta_S(\mu_S) > \beta_S(\mu_R)$. Therefore, $\det(\mathcal{K}) = \varphi(1-\varphi)\left(\beta_R(\mu_R)\beta_S(\mu_S) - \beta_R(\mu_S)\beta_S(\mu_R)\right) > 0$. Then, solving system (E.12) for (X, Y) yields to

$$\begin{cases} X = & \dfrac{\beta_S(\mu_S) - \beta_S(\mu_R)}{c_0\Lambda\varphi\left(\beta_R(\mu_R)\beta_S(\mu_S) - \beta_R(\mu_S)\beta_S(\mu_R)\right)} > 0, \\ Y = & \dfrac{\beta_R(\mu_R) - \beta_R(\mu_S)}{c_0\Lambda(1-\varphi)\left(\beta_R(\mu_R)\beta_S(\mu_S) - \beta_R(\mu_S)\beta_S(\mu_R)\right)} > 0. \end{cases}$$

Since $\mathcal{R}_0^k(x) = \frac{\varphi_k\Lambda}{\theta}\Psi_k(x) = \frac{\varphi_k\Lambda c_0(x)}{\theta}\beta_k(x)$, the above system rewrites

$$\begin{cases} \theta X = & \dfrac{\mathcal{R}_0^S(\mu_S) - \mathcal{R}_0^S(\mu_R)}{\mathcal{R}_0^R(\mu_R)\mathcal{R}_0^S(\mu_S) - \mathcal{R}_0^R(\mu_S)\mathcal{R}_0^S(\mu_R)} > 0, \\ \theta Y = & \dfrac{\mathcal{R}_0^R(\mu_R) - \mathcal{R}_0^R(\mu_S)}{\mathcal{R}_0^R(\mu_R)\mathcal{R}_0^S(\mu_S) - \mathcal{R}_0^R(\mu_S)\mathcal{R}_0^S(\mu_R)} > 0. \end{cases}$$

Coming back to the definition of $X = X(a_S, a_R)$ and $Y = Y(a_S, a_R)$ provided by (E.11), we then find

$$\mathcal{G}(a_R, a_S)^T = \left(\frac{1}{X} - \theta, \frac{1}{Y} - \theta\right)^T, \tag{E.13}$$

with $\mathcal{G} = \begin{pmatrix} \beta_R(\mu_R) & \beta_R(\mu_S) \\ \beta_S(\mu_R) & \beta_S(\mu_S) \end{pmatrix}$. Because $\det(\mathcal{G}) = \left(\beta_R(\mu_R)\beta_S(\mu_S) - \beta_R(\mu_S)\beta_S(\mu_R)\right) > 0$, it comes that for the equilibrium to be dimorphic it is necessary that

$$\begin{cases} \theta X < 1, \\ \theta Y < 1, \\ \beta_S(\mu_S)\left(\dfrac{1}{\theta X} - 1\right) > \beta_R(\mu_S)\left(\dfrac{1}{\theta Y} - 1\right), \\ \beta_R(\mu_R)\left(\dfrac{1}{\theta Y} - 1\right) > \beta_S(\mu_R)\left(\dfrac{1}{\theta X} - 1\right). \end{cases} \tag{E.14}$$

This heuristic condition (E.14) is necessary (but not sufficient) for system (2) (here with $N_c = 2$) to admit an endemic dimorphic equilibrium. The situation with a technical assumption on disjoint supports of β_k, is rigorously studied in Burie *al.* (2019).

But here, in order to go slightly further in our analysis, we assume a strong trade-off on infection efficiency, namely

$$\Psi_l(\mu_k) < 1 \text{ and } \beta_l(\mu_k) \ll 1 \text{ for } l, k = R, S \text{ and } l \neq k.$$

We deduce that the above system of equation roughly simplifies into

$$\begin{cases} \dfrac{\varphi\Psi_R(\mu_R)}{\theta + a_R\beta_R(\mu_R)} \approx \dfrac{1}{\Lambda}, \\ \dfrac{(1-\varphi)\Psi_S(\mu_S)}{\theta + a_S\beta_S(\mu_S)} \approx \dfrac{1}{\Lambda}. \end{cases}$$

Hence the proportions of each phenotype, μ_S and μ_R, can be calculated as

$$a_R \approx \frac{\varphi\Lambda\Psi_R(\mu_R) - \theta}{\beta_R(\mu_R)} \quad \text{and} \quad a_S \approx \frac{(1-\varphi)\Lambda\Psi_S(\mu_S) - \theta}{\beta_S(\mu_S)}, \tag{E.15}$$

provided the following threshold conditions in this strong trade-off framework

$$\varphi\frac{\Lambda}{\theta}\Psi_R(\mu_R) > 1 \text{ and } (1-\varphi)\frac{\Lambda}{\theta}\Psi_S(\mu_S) > 1.$$

Quantitative resistance impacting total sporulation production p_S and p_R (SP scenario). In this case, using the same argument as in Djidjou-Demasse et *al.* (2017) we can prove that the spore population is monomorphic at equilibrium such that $A(x) = a^*\delta_{\mu^*}(x)$; with $a^* > 0$, providing that we are not in a strict symmetric configuration of the fitness function. Applying the same arguments as in the previous section leads to

$$\frac{a^*}{\theta + a^*\beta(\mu^*)} m_\varepsilon(\mu^* - x)\Psi(\mu^*) = \frac{a^*}{\Lambda}\delta_{\mu^*}(x).$$

Again with $\varepsilon \to 0$, it comes

$$a^* = \frac{\Lambda\Psi(\mu^*) - \theta}{\beta(\mu^*)} = \frac{\theta}{\beta(\mu^*)}\left[\mathcal{R}_0(\mu^*) - 1\right],$$

with $\mathcal{R}_0(\mu^*) > 1$.

References

Burie, J. B., Ducrot, A., Griette, Q., Richard, Q. (2019). Concentration estimates in a multi-host epidemiological model structured by phenotypic traits. *Journal of Differential Equations*, 269(12), 11492-11539.

Djidjou-Demasse, R., Ducrot, A., Fabre, F. (2017). Steady state concentration for a phenotypic structured problem modeling the evolutionary epidemiology of spore producing pathogens. *Mathematical Models and Methods in Applied Sciences*, 27, 385-426.

F $\mathcal{R}_0$ as the fitness proxy

By Equations (D.6) and (D.7), it comes

$$f_z(y) = \sum_{k=1}^{N_c} \frac{\Lambda\varphi_k\Psi_k(y)}{\theta + \beta_k(z)A^z} - 1. \tag{F.16}$$

Using Equation (D.8) defining the resident equilibrium, i.e. $\mathcal{R}(z, E^z) = 1$, (F.16) becomes

$$f_z(y) = \sum_{k=1}^{N_c} \frac{\Lambda\varphi_k}{\theta + \beta_k(z)A^z}\left(\Psi_k(y) - \Psi_k(z)\right). \tag{F.17}$$

When infection efficiencies do not differ between host classes (*i.e.* $\beta_k = \beta(x)$, for every k and every x). Then (F.17) gives

$$f_z(y) = \frac{\theta}{\theta + \beta(z)A^z}\left(\mathcal{R}_0(y) - \mathcal{R}_0(z)\right),$$

and then

$$\text{sign}(f_z(y)) = \text{sign}\left(\mathcal{R}_0(y) - \mathcal{R}_0(z)\right). \tag{F.18}$$
